# Long‐term impact of exposure to Royal Guard, a pyriproxyfen‐based bed net, on pyrethroid‐resistant malaria vectors from Cameroon using DNA‐based metabolic resistance markers

**DOI:** 10.1002/ps.8615

**Published:** 2025-01-23

**Authors:** Emilie S Ngongang‐Yipmo, Magellan Tchouakui, Benjamin D Menze, Riccado F Tiomela, Derrick Fofie, Vanessa B Ngannang‐Fezeu, Jean L Mugenzi, Flobert Njiokou, Charles S Wondji

**Affiliations:** ^1^ Centre for Research in Infectious Diseases (CRID) Yaoundé Cameroon; ^2^ Parasitology and Ecology Laboratory, Department of Animal Biology and Physiology, Faculty of Science University of Yaoundé 1 Yaoundé Cameroon; ^3^ Department of Vector Biology Liverpool School of Tropical Medicine Liverpool UK; ^4^ International Institute of Tropical Agriculture (IITA) Yaoundé Cameroon

**Keywords:** *Anopheles*, metabolic resistance, DNA‐based markers, Royal Guard, life‐traits, longevity, fertility

## Abstract

**BACKGROUND:**

Escalating pyrethroid resistance in malaria vectors highlights the urgency of implementing new control tools incorporating non‐pyrethroid molecules. Here, using DNA‐based metabolic resistance markers, we assessed the efficacy of the dual active ingredients net Royal Guard against pyrethroids‐resistant malaria vectors in Cameroon, establishing its long‐term impact on mosquitoes' life traits after exposure.

**RESULTS:**

Cone assays revealed low efficacy of Royal Guard against field *Anopheles* populations. However, analysis of the survival curves revealed that unexposed mosquitoes lived longer (11.4 ± 0.4 days) than those exposed to Royal Guard (7.9 ± 0.2 days) (*χ* ^2^ = 6; *P* = 0.05), indicating that despite the lower immediate mortality observed against resistant mosquitoes, there is a long‐term effect on *Anopheles funestus* longevity. High blood‐feeding inhibition rate was observed (44–80% *versus* 11–41%), indicating that this net has a negative impact on blood meal intake. Additionally, female mosquitoes exposed to this net exhibited a 25% reduction in oviposition, 18.30% reduction in fecundity, 8.10% reduction in offspring, and a 74.74% infertility rate compared to the control. Genotyping of key resistance markers revealed that, metabolic markers and L1014F*‐Kdrw* are associated with the reduced efficacy observed, with homozygote‐resistant mosquitoes significantly more able to survive and live longer after exposure than homozygote‐susceptible mosquitoes (odds ratio = 15.79; confidence interval = 5.35–43.27; *P* < 0.0001).

**Conclusion:**

This study revealed that although pyrethroid‐resistant mosquitoes have higher ability to survive and live longer after exposure to Royal Guard, this net significantly affects their lifespan, blood‐feeding ability and interestingly reduces their fecundity/fertility. © 2025 The Author(s). *Pest Management Science* published by John Wiley & Sons Ltd on behalf of Society of Chemical Industry.

## Graphical abstract

We used recently detected P450 DNA‐based markers of pyrethroid resistance in both *Anopheles funestus* and *Anopheles gambiae* to demonstrate that although pyrethroid‐resistant mosquitoes have higher ability to survive and live longer after exposure to Royal Guard, this net significantly affects their lifespan, blood feeding ability and interestingly reduces their fecundity/fertility.

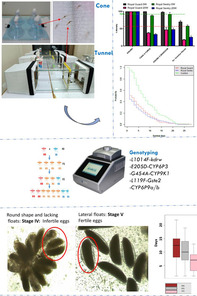

## 1 BACKGROUND

Malaria prevention largely depends on the control of mosquitoes using insecticide‐based interventions such as long‐lasting insecticide‐treated nets (LLINs) and indoor residual spraying (IRS).^1^ Pyrethroids are the primary class of insecticide recommended for LLINs treatment, while organochlorines, organophosphates, carbamates, and pyrethroids and new families of insecticides (pyrrole and neonicotinoid), are currently available for IRS.^2^

The use of the earlier‐mentioned insecticides over the past years in agricultural and/or public health sector has led to the development of insecticide resistance in mosquitoes,^3^ reducing the effectiveness of pyrethroid‐only nets. Escalation of pyrethroid resistance encouraged manufacturers to develop new types of LLINs as part of the Global Plan for Insecticide Resistance Management in malaria vectors (GPRIM).^4^ Efforts to maintain the efficacy of bed nets in areas of pyrethroid resistance have driven the development of alternative net types containing either insecticide synergists [piperonyl butoxide (PBO)‐based nets] and recently dual active ingredient (AI) nets, including nets combining pyrethroid plus insect growth regulators (IGRs).^4^ Establishing the performance of these ‘new generation nets’ against pyrethroid resistant vectors is essential for future control and elimination of malaria.^5^

IGRs for example are insecticides with high selectivity, and they interfere with insect growth, development, and metamorphosis and consequently reduce mosquitoes density.^6^ IGRs are safe for the environment and non‐target organisms, including mammals.^7^ Three major groups of IGRs are well documented including the juvenile hormone analogues (JHAs), the ecdysone agonists, and the chitin synthesis inhibitors.^8^ JHAs are relatively under‐exploited class of insecticides.^9^ This group contains pyriproxyfen (PPF) which is a synthetic juvenile hormone (JH) mimically used as a larvicide that interferes with the development of mosquito larvae preventing the emergence of adults.^10^ PPF inhibits mosquito metamorphosis and has been used for decades against cotton pests.^11^ Due to its effectiveness at low concentrations, PPF can be spread by the insect itself, which has been effective in controlling *Aedes* populations in Peru.^12^

Because PPF induces sterilization in adult mosquitoes,^13^ mixing it in a LLIN formulation with a pyrethroid has the potential to prevent the spread of pyrethroid‐resistant mosquitoes.^6^ Royal Guard is a recent pyrethroid‐PPF based net developed by Disease Control Technologies, Greenville, SC, USA. It is treated with a mixture of alpha‐cypermethrin and PPF, both incorporated into the monofilament yarn. Exposure to PPF has been studied extensively in *Aedes* mosquitoes^14^ and previous studies demonstrated that PPF‐treated bed nets have shown promise in suppressing mosquito reproduction and reducing mosquito populations under semi‐field conditions.^15^ Moreover, Royal Guard nets were shown to be effective against pyrethroid resistance nets using experimental huts in some parts of Africa^16^ and relatively more effective than pyrethroid‐only nets in a randomized controlled trial in Benin in the background of a resistant population.^17^

As this new net is recommended for use on a large scale, it is vital to assess the impact of exposure to these LLINs on the life traits of the vectors and to evaluate the impact of known resistance markers on the ability to lay eggs, to blood feed and to live for a long time. The recent design and establishment of several metabolic resistance markers (detoxification genes: *CYP6P9a/b*, 6.5Kb‐SV, *CYP9K1*, *GSTe2* and *CYP6P3*) combined with the knockdown target site resistance (*Kdr*‐*W*) marker provides an excellent opportunity to establish such impact. It was shown for example that PermaNet 2.0 presents a reduced efficacy against resistant populations but remains efficient after exposure by reducing the life expectancy of the vectors.^18^, ^19^ However, the impact of key resistance mechanisms to pyrethroid on the effectiveness of Royal Guard remains uncharacterized, notably in the context of the long‐term consequences on mosquitoes' fitness and reproduction.

Here, we investigated the efficacy of Royal Guard and the post‐exposure effect on different life trait parameters (blood feeding, fecundity, fertility, and longevity) under laboratory conditions and the effect of key resistance markers. The results revealed that beyond the immediate effect, Royal Guard has a long‐term negative impact on life traits of mosquitoes with significantly reduced longevity and fertility compared to controls. Genotyping of DNA‐based resistance markers showed that metabolic resistance combines with the target‐site resistance to exacerbate the loss of efficacy of Royal Guard while reducing the long‐term impact of exposure to this net.

## 2 METHODS

### 2.1 Study sites

Mosquitoes used in this study were collected from two sites in Cameroon including, Elendé (3°41′57.27″ N, 11°33′28.46″ E) a rural village situated in the central region and close to the capital city of Yaoundé and Mangoum (5°29′09.2″ N, 10°35′20.8″ E) a village located in the western part of the country and characterized by extensive agricultural practices. Elendé is highly endemic (792 infective bites/person/year) to malaria mainly driven by *Anopheles funestus* s.s.^20^ In Mangoum, *Anopheles gambiae* s.s. is the predominant malaria vector with 4.1% plasmodium infection rate. This species is resistant to the four main classes of public health insecticides, with mortality rates < 50% at ten times (10×) the pyrethroid discriminating doses.^21^

### 2.2 Collection and rearing of field mosquitoes

Both *An. funestus* s.l. from Elendé and *An. gambiae* s.l. (females) from Mangoum were sampled at the adult stage (indoor resting female) using electric aspirators. They were forced to lay eggs and larvae were reared till the adult stage (F_1_) at the insectary of the Centre for Research in Infectious Diseases (CRID), Yaoundé, Cameroon. Two‐ to five‐day‐old female mosquitoes (F_1_) from the collected adults (F_0_) were used for cone assays. The good distribution of the G454A‐*CYP9K1* and L119F‐*GSTe2* markers in *An. funestus* from Elendé facilitated the use of these resistance markers for the molecular analysis.

### 2.3 Maintenance of susceptible laboratory strains

In field populations of malaria vectors, most of the insecticide resistance markers are already fixed hindering the possibility of establishing any phenotype–genotype correlation. To overcome this challenge, two reciprocal crosses between field resistant and laboratory susceptible mosquitoes were established at the insectary of the CRID. A crossing between KISUMU (the susceptible strain) and field‐resistant mosquitoes collected from Mangoum for *An. gambiae*. For *An. funestus*, a crossing was done between FANG, a fully susceptible laboratory strain colonized from Angola^22^ and FUMOZ‐R, a pyrethroid‐resistant strain homozygous resistant for two major cytochrome P450 markers *CYP6P9a* and *CYP6P9b* ^23^, ^24^ originating from Mozambique.^25^ To perform the crosses, pupae of each strain were collected and put individually into hemolysis tubes for individual emergence, after which males of the resistant strain were mixed in the same cage with females of the susceptible colony for random mating to generate the first generation (F_1_).

All mosquitoes were reared under standard insectary conditions of 27 ± 2 °C and 80 ± 10% relative humidity and the bioassays were performed using F_3_‐F_4_ offspring for *An. funestus* hybrids (FANG × FUMOZ‐R) allowing the genotyping of *CYP6P9a/b* and 6.5Kb‐SV and F_3_ for *An. gambiae* hybrids (KISUMU × MANGOUM) allowing the genotyping of *CYP6P3* and *Kdr‐W*.

### 2.4 Brand name of the nets used

Three brands of LLINs sourced directly from the manufacturer were tested (0 wash and 20 times‐washed).

1. Control or untreated (bed nets without insecticide)
2. Royal Sentry®: alpha‐cypermethrin 203 mg/m^2^
3. Royal Guard®: 208 mg/m^2^ alpha‐cypermethrin +208 mg/m^2^ PPF

### 2.5 Washing procedure of nets

To prepare the washing solution, 20 g of Marseille soap was added to 10 L of dechlorinated water (2 g/L) in an aluminium washing tank. Each net was immersed in a separate washing solution for 10 min. During the washing, the net underwent two manual rotation periods of 20 rpm for 3 min, interspersed with 4 min of holding. The net was rinsed following the same steps as the washing using only dechlorinated water and then dried out in the sun.

### 2.6 Evaluation of the bio‐efficacy of Royal Guard on wild populations of *An. funestus* and *An. gambiae* in WHO cone bioassays

Five 25 cm × 25‐cm squares were cut from each net, wrapped in aluminum foil, and kept at 4 °C until the insecticide activity tests were performed. The susceptible laboratory strain of *An. gambiae* s.s. (KISUMU) was used to first assess the quality control of the unwashed and washed nets. After quality control, wild populations of *An. gambiae* s.l. (Mangoum, Nkolondom, Gounougou, Elendé) and *An. funestus* (Elendé, Mibellon) collected across Cameroon beside laboratory and hybrid colonies (FUMOZ‐R, FUMOZ × FANG and KISUMU × MANGOUM hybrids) were tested to establish the performance of the PPF‐LLIN Royal Guard on local pyrethroid resistant mosquitoes in comparison to the pyrethroid‐only net Royal Sentry.

Cone bioassays were performed with Royal Guard and Royal Sentry unwashed (Royal Guard 0W and Royal Sentry 0W) and 20‐times washed (Royal Guard 20W and Royal Sentry 20W) according to standard World Health Organization (WHO) procedures.^1^ Mosquitoes were exposed to the insecticide nets for 3 min using plastic cones, then were gently but rapidly removed from the cones using a mouth aspirator and transferred into paper cups. Knockdown was recorded 60 min after exposure. Alive mosquitoes after exposure were fed with a 10% sugar solution on soaked cotton balls and mortality was recorded 24 h post‐exposure. The impact of exposure on different life traits of the vector was established focusing on blood feeding, fecundity, fertility, and longevity. All experiments were carried out at an ambient temperature of 25 °C ± 2 °C and 80 ± 10% relative humidity.

### 2.7 Tunnel test bioassays

Tunnel tests were performed for Royal Guard in addition to the cone assay to evaluate the efficacy and the personal protection. A total of 100 sugar‐starved (for 1 h) mosquitoes aged 5–8 days were released in the long section of the glass tunnel at 06:00 p.m. A guinea pig was used as bait and positioned on the other side of the net so that mosquitoes could pass through the holed net to access the bait and feed. The following morning, between 06:00 and 09:00 a.m., mosquitoes were removed (separately from each section of the tunnel) using a mouth aspirator, counted, and scored as alive or dead, blood‐fed or unfed after which they were held for 72 h with access to 10% sugar solution at 27 °C ± 2 °C and 80 ± 10% relative humidity. The main outcome measures were 12 h mortality measured in the morning after the experiment, 24 h post‐exposure mortality, and post‐exposure effect of blood feeding inhibition.^1^

### 2.8 Sterilizing properties of PPF using CDC bottle bioassays

Briefly, 1 day before exposure, a solution of PPF (Sigma‐Aldrich, St Louis, MO, USA) was prepared by adding 0.005 g to 50 mL of 100% acetone. Four bottles were then coated with 1 mL of acetone (for control bottles), others with 1 mL of PPF solution of a concentration of 100 μg and with alpha‐cypermethrin (12.5 ug to 50 mL of acetone). Each bottle was labelled (name, insecticide concentration, date), wrapped in aluminum foil, and left open to dry in an air conditioned room with a temperature between 20 and 25 °C. Blood‐fed females were introduced in batches of 25 for 1 h exposure in coated bottles (control, PPF, and alpha‐cypermethrin). They were then transferred back into labelled holding paper cups for 72 h and fed with 10% sugar solution, the number of live and dead mosquitoes was recorded every 24 h. The mosquitoes were dissected to evaluate the sterilizing effect of PPF on their ovaries.

### 2.9 Assessing the long‐term impact of exposure to Royal Guard on key life traits of mosquitoes

#### 2.9.1 Evaluating the effect of Royal Guard on blood feeding ability after exposure

Two hybrid mosquito strains FANG × FUMOZ and KISUMU × MANGOUM were used to evaluate the impact of Royal Guard exposure on the ability to take a blood meal. Each strain was first exposed to the net (Royal Guard 20W, Royal Sentry 20W and control) for 3 min, and after 24 h holding period, the alive were removed and introduced to the new paper cups and blood‐fed for 5 min. At the end of the experiment,^26^ the number of blood‐fed and unfed mosquitoes was recorded then, the blood feeding rate was compared between different treatments. Similar comparisons were done after the tunnel test. A subset of blood fed and unfed mosquitoes were genotyped for key markers of resistance to establish the association between the presence of these markers and the ability of mosquitoes to take a blood meal.

#### 2.9.2 Assessing the impact of Royal Guard exposure on fecundity and fertility: oviposition inhibition, reduction in fecundity and reduction in offspring

One batch of mosquitoes from the three experimental groups were blood‐fed twice 48 h post‐exposure and kept for 4 days to become fully gravid. Gravid females were then put individually in 1.5 mL Eppendorf tubes with damp filter paper to allow them to lay eggs as previously described.^27^ The number of eggs laid per female and the number of larvae obtained after hatching were counted and recorded.^28^ Different indices (reduction in oviposition rate, reduction in fecundity and reduction in offspring) were assessed to evaluate the sterilizing effect of PPF impregnated on Royal Guard compared to the standard net Royal Sentry and the untreated net. As indicated earlier, a subset of oviposited and non‐oviposited mosquitoes were genotyped for key markers of resistance to establish the association between the presence of these markers and the ability of mosquitoes to lay eggs.

#### 2.9.3 Physiological impact of PPF on ovary development

Batches of hybrid mosquitoes from the control, Royal Guard 20W, and Royal Sentry 20W groups in cone test and/or CDC bottle were dissected 4 days after blood meal to look at the impact of PPF (JH) on ovary development. Females were anesthetized on ice for 5 min and dissected under a microscope (Leica DM750; Leica, Wetzlar, Germany) at 200× magnification. A fine‐scale observation was done at 400× magnification for a focused view of egg development. Images of the ovaries were taken using a digital camera. Mosquito egg stages were classified according to the Christopher's stages as follows.^29^

- Fertile: eggs of female mosquitoes fully developed to Christopher's stage V (normal, boat/sausage‐shaped eggs with floats);
- Infertile: eggs of female mosquitoes not fully developed and remain in Christopher's stages I–IV (less elongated, round eggs without floats).

#### 2.9.4 Evaluating the effect of Royal Guard on longevity after exposure

To evaluate the longevity after exposure to the bed net, we used the two hybrid strains (FANG × FUMOZ and KISUMU × MANGOUM) and field strains: *An. funestus*, *An. gambiae*. After exposing the different strains to Royal Guard 20W, Royal Sentry 20W and control net, alive females from each group were collected and transferred into different cups. Dead mosquitoes were counted each morning and removed from the cups and those alive were counted and fed with a 10% sucrose solution every day (from the beginning to the end of the experiment in each cup). The mortality curves were compared between the treated group and untreated mosquitoes. Genotyping of some resistant markers was done to assess the association with life span and potential increased ability to survive after exposure to the insecticide. All the mosquitoes were genotyped for key markers of resistance and grouped by intervals of days to establish the association between the presence of these markers and the ability of mosquitoes to live long.

### 2.10 Assessing the impact of pyrethroid‐resistant markers on mosquito's ability to withstand Royal Guard exposure

Key resistance markers including L1014F*‐Kdr‐*W and E205D*‐CYP6P3* of *An. gambiae* and G454A*‐CYP9K1* and L119F*‐GSTe2* of *An. funestus* were genotyped in field mosquitoes (alive *versus* dead post‐exposure to the nets, oviposited *versus* non‐oviposited and, blood‐fed *versus* unfed) to establish the statistical significance of any association between these pyrethroid resistance markers and the various life traits parameters. Similar analysis was carried out for the *CYP6P9a/b* and *6.5Kb‐SV* markers in the hybrid laboratory strain FANG × FUMOZ. The genotyping was performed using previously established protocol.^23^, ^24^, ^30^, ^31^, ^32^, ^33^ All primer sequences are listed in Supporting Information Table S2.

#### 2.10.1 Expression profile of reproductive genes after pyriproxyfen exposure using qRT‐PCR

The quantitative reverse‐transcription polymerase chain reaction (qRT‐PCR) was performed to assess the expression level of five reproductive genes implicated in the ovogenesis process: Methroprene tolerant (Met), Krüppel homologue 1 (Kr‐h1), Ecdysone receptor A (EcRA), Hormone receptor 3 (HR3) and 20‐hydroxyecdysone (20E). Gene‐specific primers are listed in Table S1. Total RNA from pools of 25 ovaries from KISUMU (full susceptible strain) and the hybrid strain KISUMU × MANGOUM exposed to acetone and PFF was extracted using the Arcturus Picopure RNA Isolation Kit (Life Technologies, Carlsbad, CA, USA). Briefly, 1 μg of RNA from each group, was used as a template for complementary DNA (cDNA) synthesis using the superscript III (Invitrogen, Carlsbad, CA, USA) with oligo‐dT20 and RNase H, following the manufacturer's instructions. The qRT‐PCR was carried out using three biological replicates in an MX3005 real‐time PCR system (Agilent, Santa Clara, CA, USA) using Brilliant III Ultra‐Fast SYBR Green qPCR Master Mix (Agilent) as described previously.^34^, ^35^ The relative expression level and fold‐change (FC) of reproductive genes relative to the susceptible strain was calculated according to the 2^−ΔΔCT^ method^36^ after normalization with the housekeeping genes ribosomal protein S7 (RSP7) and Elongation factor (EF).

### 2.11 Data analysis

The mortality rate for each test, following a 24‐h holding period after a 3 min exposure to LLINs was calculated using Microsoft Excel 2010 (Microsoft Corp., Redmond, WA, USA). The efficacy of standard and dual AI nets was evaluated by comparing the mean mortality rate obtained between different brand nets using the Chi‐square test (*χ* ^2^). Survival curves were estimated using the Kaplan–Meier estimator and compared using the non‐parametric Tarone–Ware test. Fisher's exact test and odds ratio (OR) were then used to establish the statistical significance of any association between resistant markers and the ability of mosquitoes to survive bed net exposure. Additionally, these statistical tests were used to compare the effect of the resistant marker on delayed mortality by comparing the frequency of resistant alleles between mosquitoes at different time points, between blood‐fed females *versus* unfed, and between oviposited females *versus* non‐oviposited females. Graphs were constructed using R version 4.3.3 software and GraphPad Prism version 7.00 (GraphPad Software Inc., San Diego, CA, USA). Various indices were assessed to establish the sterilizing effect of the Royal Guard net compared to the standard Royal Sentry net and the untreated net.

The following outcomes were used to assess the reproductive effects on surviving female mosquitoes.^16^

1. *Proportion of reduction in oviposition rate*

The reduction in the proportion of females ovipositing for a given treatment compared to the control. This was calculated as follows:

$$\frac{100\left(\mathrm{Oc}-\mathrm{Ot}\right)}{\mathrm{Oc}}$$

where Oc is the proportion of surviving blood‐fed females from the control which laid eggs and Ot is the proportion of surviving blood‐fed females from a given treatment which laid eggs.

2 *Proportion of reduction in fecundity*

The reduction in the number of eggs per surviving blood‐fed female for a given treatment relative to the control. This was calculated as follows:

$$\frac{100\left(\mathrm{Ec}-\mathrm{Et}\right)}{\mathrm{Ec}}$$

where Ec is the mean number of eggs per surviving blood‐fed female observed in the control while Et is the mean number of eggs per surviving blood‐fed female observed in a given treatment.

3 *Proportion of reduction in offspring*

The percentage reduction in the number of larvae per surviving blood‐feed female observed for a given treatment relative to the control. This was calculated as follows:

$$\frac{100\left(\mathrm{Lc}-\mathrm{Lt}\right)}{\mathrm{Lc}}$$

where Lc is the mean number of larvae per surviving blood‐fed female observed in the control while Lt is the mean number of larvae per surviving blood‐fed female observed in a given treatment.

## 3 RESULTS

### 3.1 Bio‐efficacy of insecticide‐treated nets against field resistant mosquitoes using cone and tunnel tests

**Figure 1:**
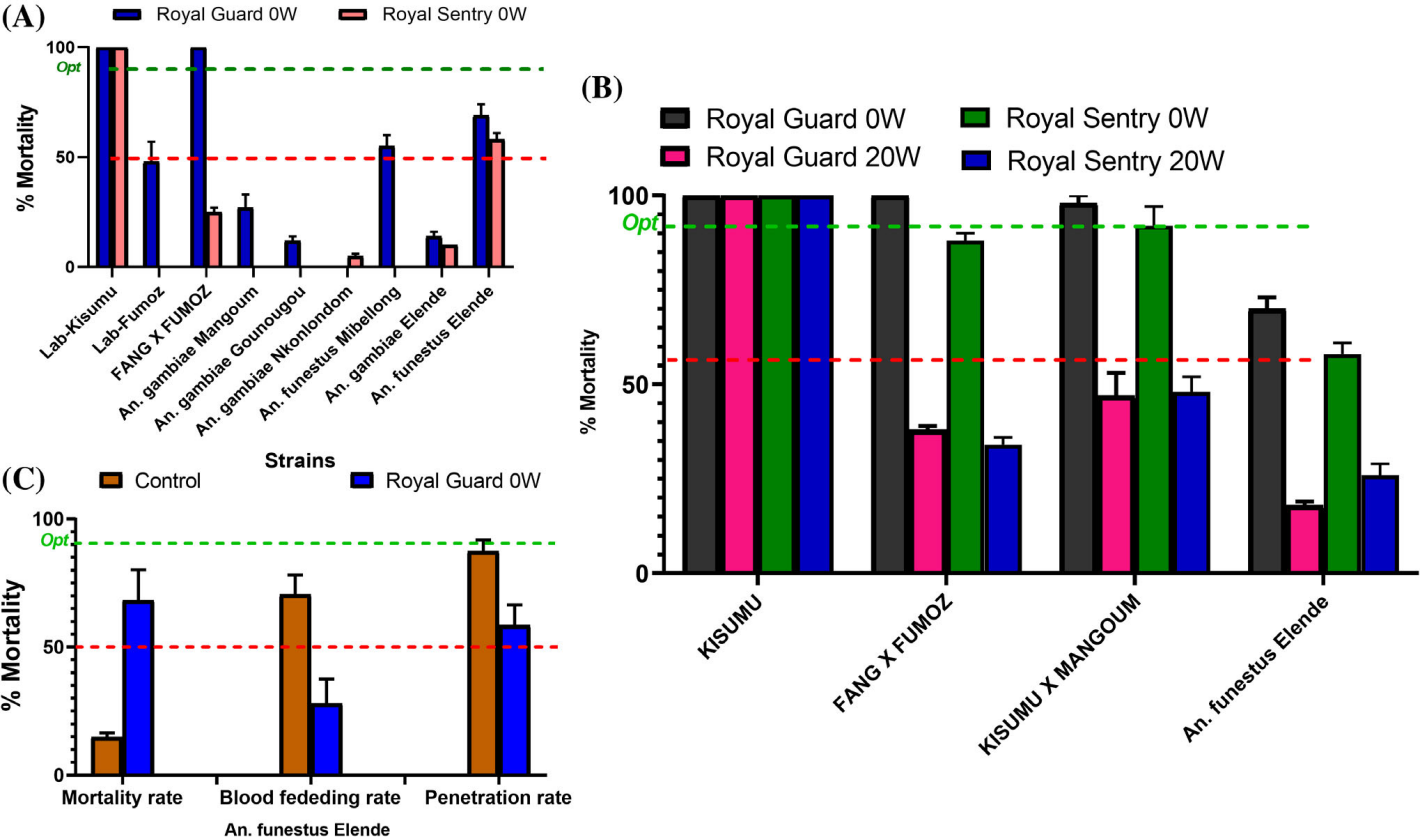
Performance of Royal Guard and Royal Sentry against *Anopheles gambiae* s.l. and *Anopheles funestus* mosquitoes. (A) Mortality rate 24 h after 3‐min cone test for the two nets against different wild *Anopheles* populations. (B) Mortality rate after 3‐min cone test with Royal Guard 0W and 20W for hybrids and (C) for field *An. funestus* after tunnel test of wild *An. funestus* against Royal Guard. Red dotted line represents minimal efficacy (50% mortality) and the green dotted line represents optimal efficacy (80% mortality) of the nets. Error bars represent the standard error on the mean (SEM); 0W, unwashed nets; 20W, 20 times‐washed nets.

Field populations of *An. gambiae* mosquitoes tested showed a very low mortality against the unwashed Royal Guard and Royal Sentry (0–17%) (Fig. 1(A)). However, a moderate mortality was observed for *An. funestus* from Mibellon (55%) and Elendé (69%) (Fig. 1(A)). Both hybrid strains used in this study revealed higher mortality against the unwashed dual AI net Royal Guard (> 98% mortality). Interestingly, the 20‐times washed Royal Guard induced 100% mortality on the susceptible laboratory strain KISUMU in cone assay (Fig. 1(B)). However, reduced performance was observed after exposure to 20 washes net compared to unwashed for FANG × FUMOZ (100% mortality for 0 washes and 38% over 20 washes), same for KISUMU × MANGOUM (98% mortality for 0 washes and 47% over 20 washes). Similarly, results were obtained for Royal Sentry net (Fig. 1(B)). A reduction of mortality was observed with the field *An. funestus* from Elendé (70% mortality for 0 washes and 18% over 20 washes) (Fig. 1(B)) indicating that after 20 washes, the net is still efficient on the susceptible laboratory strain but loses its performance against pyrethroid resistant strain. After the tunnel test with wild *An. funestus*, the PPF‐based net Royal Guard induced a higher mortality (68%), reduced the blood‐feeding rate (28%) and the penetration rate (58%) compared to the control (Fig. 1(C)).

### 3.2 Long‐term impact of exposure to the PPF‐based net Royal Guard on mosquitoes' life traits

Because of the higher mortality rate obtained with the unwashed Royal Guard on many strains, 20‐times washed nets were used for post‐exposure studies because of the need of having the alive to assess the impact of exposure on the life‐traits.

#### 3.2.1 Effect on blood feeding

**Figure 2:**
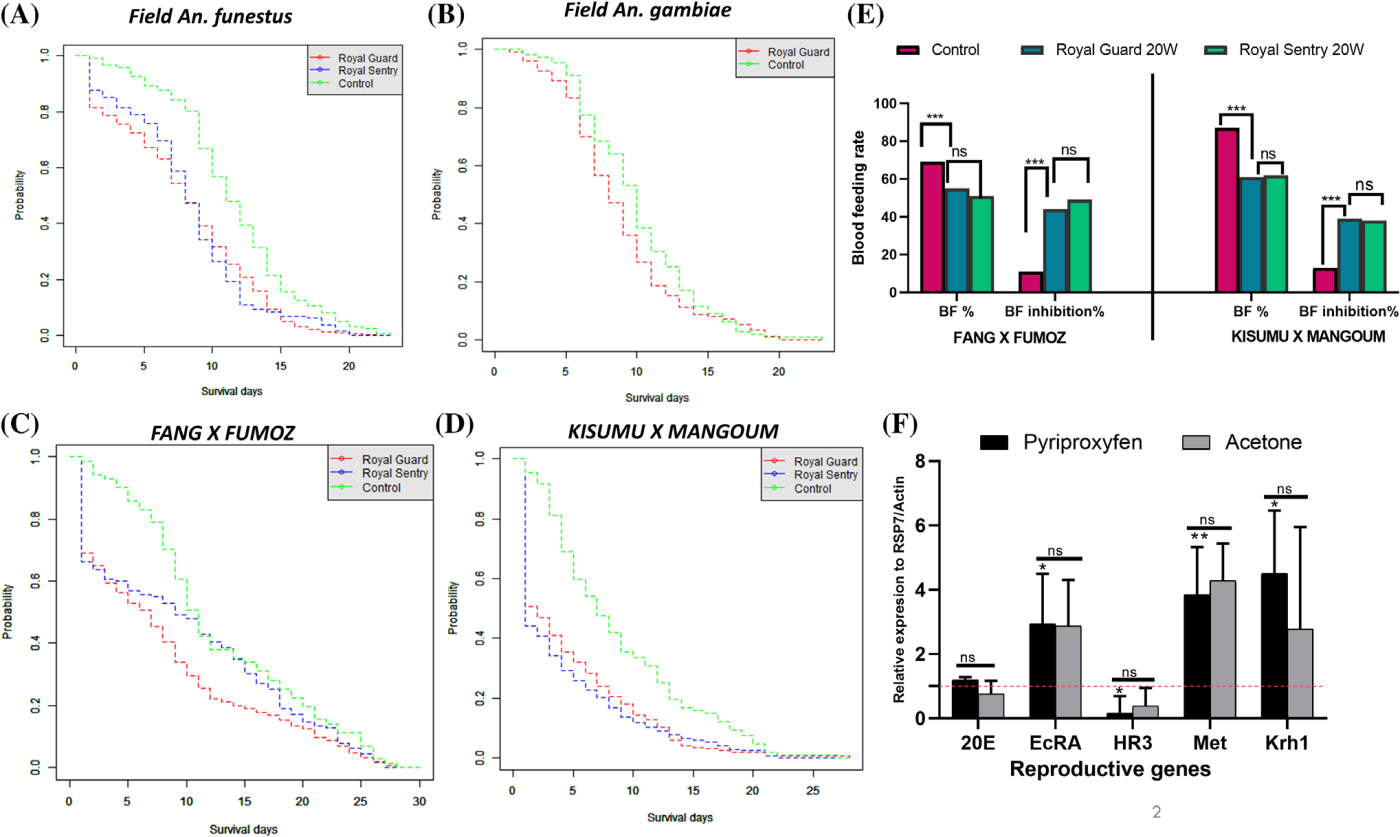
Effect of Royal Guard exposure on different life traits. Delayed mortality post‐exposure of field *Anopheles funestus* strain (A), field *Anopheles gambiae* (B), hybrids FANG × FUMOZ (C) and KISUMU × MANGOUM (D). (E) Blood‐feeding inhibition after exposure to Royal Guard and Royal Sentry. (F) Effect of pyriproxyfen exposure on the expression level of reproductive genes. Asterisk (*) represents the level of significance for each LLIN relative to the control net and between the two nets; ns, non‐significant difference compared to the control or between the two nets; 0W, unwashed nets; 20W, 20 times‐washed nets. Fold changes of messenger RNA abundance in the ovaries of PPF‐treated mosquitoes were determined relative to the acetone control group and to the susceptible strain KISUMU. Data are presented as mean ± standard deviation from three independent replicates. Statistical analysis was performed using a paired *t*‐test (ns, non‐significant difference compared to the control; *, *P* < 0.05; **, *P* < 0.01; ***, *P* < 0.001).

After cone and tunnel tests, the blood feeding rate of the hybrid FANG × FUMOZ and KISUMU × MANGOUM relative to the control, was higher for the dual net Royal Guard (*χ* ^2^ = 7.2, *P* = 0.007); (*χ* ^2^ = 8.7, *P* = 0.003) respectively (Fig. 2(E)). Similar blood feeding rates were found with the standard net Royal Sentry (*χ* ^2^ = 7.6, *P* = 0.005); (*χ* ^2^ = 9.7, *P* = 0.001) (Fig. 2(E)) for the two hybrid strains. These results suggest that both Royal Guard and Royal Sentry net had a negative effect on female blood feeding ability. The result from the tunnel assay confirmed the previous outcomes, where after a longer exposure time with Royal Guard, the blood feeding inhibition was significantly higher compared to the control (*χ* ^2^ = 76.3, *P* < 0.0001) for FANG × FUMOZ (Supporting Information, Fig. S1).

#### 3.2.2 Effects of PPF‐based net Royal Guard on fecundity and fertility

Table 1 presents the effect of the different net brands on the fecundity and fertility of blood fed hybrid mosquitoes that survived Royal Guard exposure, compared to Royal Sentry. For hybrid *An. funestus* strain FANG × FUMOZ, the oviposition rate was significantly higher for the control net (*χ* ^2^ = 3.6, *P* = 0.005) and Royal Sentry (*χ* ^2^ = 6.2, *P* = 0.01) compared to Royal Guard showing the sterilizing effect of Royal Guard. Also, the mean number of eggs obtained was higher in the control group (mean eggs = 71 ± 2.5) compared to Royal Sentry (mean eggs = 59.6 ± 6.06), Royal Guard (mean eggs = 57.9 ± 0.8). A significant difference between the mean number of eggs from the control compared to Royal Guard (*t* = 47.6, *P* < 0.0001) and Royal Sentry (*t* = 40.3, *P* < 0.0001) was observed, indicating that exposure to a PPF‐based net significantly reduces the fecundity of the vectors. Furthermore, a significant difference was observed in the hatching rate between eggs from mosquitoes exposed to the untreated net and those exposed to Royal Guard (*χ* ^2^ = 7.9, *P* = 0.004). Exposure to Royal Guard induced 25% reductions in oviposition, 18% reductions in the fecundity and 8% reduction in offspring in mosquitoes compared to control group indicating that even after multiple washes, the net still has sterilizing effect on female *Anopheles* mosquitoes (Table 1). A similar pattern was observed with the *An. gambiae* strain KISUMU × MANGOUM (Table 1). Overall, the mean number of eggs for the Control was 54.1 ± 10.6 eggs, 38.3 ± 12.2 for Royal Sentry and 43 ± 28.2 for Royal Guard. A significant reduction in oviposition was observed in the Royal Guard group compared to the control (78.6%). Although mosquitoes were exposed to the 20 washes nets, a significant reduction in fecundity was observed (20.5% compared to the control) showing that Royal Guard has a strong negative impact on fecundity even after 20 washes.

**Table 1:** Effects of pyriproxyfen (PPF) on hybrids *Anopheles funestus* and *Anopheles gambiae* egg laying and viable offspring production

Strains	Bednets	*N*	Mortality (%)	*N* Blood fed	Oviposition rate (%)	*N* Eggs	Mean eggs/female	*N* Larvae	Mean larvae/females	Hacthing rate (%)	Oviposition inhibition (%)	Reduction in fecundity (%)	Reduction in offspring (%)
FANG × FUMOZ	Control	83	4.81	71	41.00	1429	71	757	37	52.95	—	—	—
	Royal Sentry 20W	151	49.66	60	48.71	1132	59,57	318	16.76	28.09	5	15.49	54.05
	Royal Guard 20W	328	49.69	105	24.33	888	57,93	523	34.1	58.87	25.00	18.30	8.10
KISUMU × MANGOUM	Control	119	89.91	89	45.16	758	54.14	212	15.14	27.00	—	—	—
	Royal Sentry 20W	266	42.48	32	28.57	307	38.37	59	7.37	19.21	42	29.12	51.32
	Royal Guard 20W	213	24.41	18	33.33	129	43	30	10	23.25	78.57	20.57	33.94

### 3.3 Physiological impact of Royal Guard on ovary development

In the FANG × FUMOZ strain, all 29 females from the control group generated fully developed eggs, indicating 0% infertility. In contrast, among the 70 females exposed to Royal Guard (washed 20 times), only 18% (13/70) were infertile. These results demonstrate that, despite a significant reduction in insecticide concentration after 20 washes, the net still exerts a negative impact on female fertility. Interestingly, when mosquitoes were directly exposed to the insecticide PPF in a CDC bottle assay, a striking 74.5% (82/110) of females were infertile, highlighting the potent sterilizing effect of the PPF product (*χ* ^2^ = 40.3, *P* < 0.0001) on vectors, as illustrated in Fig. S1(A), similar sterilization effect was observed in the *An. gambiae* strain KISUMU × MANGOUM, where the infertility rate (96%) was very high after direct exposure to the PPF 100 μg in CDC bottle test compared to acetone (infertile rate: 0%) (*χ* ^2^ = 174.7, *P* < 0.0001). However, a lower impact, with 31.8% sterility, was observed when mosquitoes were exposed to the insecticide treated net Royal Guard 20W and control net (2.1% infertile rate) (*χ* ^2^ = 12.2, *P* = 0.0005) (Table 2).

**Table 2:** Summary of females dissected after exposure using FANG × FUMOZ and KISUMU × MANGOUM

Method	Strain	Insecticides	Mortality (%)	*N* Dissected	Infertile	Fertile	Infertile (%)	Fertile (%)
CDC bottles assay	KISUMU	Acetone	0	11	0	11	0	100
		PFF 100 U	0	33	33	0	100	0
	FANG × FUMOZ	Acetone	0	33	4	29	12.12	87.87
		Alpha‐Cyp 12.5 U	80.86	26	8	18	30.76	69.23
		PFF 100 U	2.67	110	82	28	74.54	25.45
	KISUMU × MANGOUM	Acetone	0	91	0	91	100	0
		Alpha‐Cyp 12.5 U	—	—	—	—	—	—
		PFF 100 U	4	100	96	4	96	4
Cone assay	FANG × FUMOZ	Control	9.74	29	0	29	0	100
		Royal Sentry 20W	50.19	26	2	24	7.69	92.3
		Royal Guard 20W	62.37	70	13	57	18.57	81.42
	KISUMU × MANGOUM	Control	17.30	46	1	45	2.17	97.82
		Royal Sentry 20W	81.00	23	6	17	26.08	73.91
		Royal Guard 20W	88.20	22	7	15	31.81	68.18

*Note*: PPF 100 U, pyriproxyfen 100 Ug; Alpha‐Cyp, alpha‐cypermethrin.

### 3.4 Effect of pyriproxyfen exposure on the expression level of reproductive genes

Evaluation of the differential expression of reproductive genes 24 h after blood feeding revealed that, exposure to PPF induces the over‐expression (FC = 4.5 ± 1.9) of the Kr‐h1, a JH‐inducible transcriptional regulator, when compared to the control (FC = 2.7 ± 3.2) (Fig. 2(F)). In contrast, PPF exposure induced a down‐regulation of key genes in the 20E regulatory cascade, including HR3 (FC = 0.2 ± 0.5), EcRA (FC = 2.9 ± 1.5) and Met (FC = 3.8 ± 1.5) at the previtellogenic stage (Table 3).

**Table 3:** Summary of average life span for each genotypes after genotyping

	CYP6P3				Kdr‐W			
	Royal Guard		Royal Sentry		Royal Guard		Royal Sentry	
Mean days	RR = 8.95 ± 5.24		RR = 7.37 ± 5.37		RR = 9.88 ± 4.65		RR = 8.16 ± 5.28	
	RS = 6.02 ± 4.94		RS = 6.24 ± 5.24		RS = 8.07 ± 5.13		RS = 5.87 ± 4.48	
	SS = 5.94 ± 6.49		SS = 6 ± 4.60		SS = 4.88 ± 4.7		SS = 4.05 ± 4.47	

	*t‐*Test	*P* Value	*t‐*Test	*P* Value	*t‐*Test	*P* Value	*t‐*Test	*P* Value
RR *versus* RS	0.10	0.91	1.42	0.15	0.07	0.94	1.07	0.28
RR *versus* SS	0.04	0.96	0.75	0.45	0.20	0.84	0.45	0.64

	CYP6P9a				CYP6P9b			
	Royal Guard		Royal Sentry		Royal Guard		Royal Sentry	
Mean days	RR = 7.52 ± 4.55		RR = 15.6 ± 6.31		RR = 5.86 ± 3.56		RR = 13.8 ± 6.05	
	RS = 5.57 ± 3.77		RS = 13.4 ± 7.61		RS = 5.3 ± 3.65		RS = 14.2 ± 7.46	
	SS = 1.53 ± 1.48		SS = 1.31 ± 1.55		SS = 1 ± 0		SS = 1.36 ± 1.6	

	*t‐*Test	*P* Value	*t‐*Test	*P* Value	*t‐*Test	*P* Value	*t‐*Test	*P* Value
RR *versus* RS	0.30	0.75	1.22	0.22	0.38	0.70	1.78	0.07
RR *versus* SS	2.41	0.01*	1.91	0.06	0.25	0.79	1.20	0.23

6.5Kb‐SV				
	Royal Guard		Royal Sentry	
Mean days	RR = 7.47 ± 4.72		RR = 14 ± 7.93	
	RS = 5.66 ± 3.77		RS = 13.5 ± 6.51	
	SS = 1.32 ± 1.23		SS = 3.08 ± 5.4	

	*t‐*Test	*P* Value	*t‐*Test	*P* Value
RR *versus* RS	0.27	0.78	1.33	0.18
RR *versus* SS	2.22	0.028	0.27	0.78

	CYP9K1				GSTe2‐L119F			
	Royal Guard		Royal Sentry		Royal Guard		Royal Sentry	
Mean days	RR = 12 ± 5.11		RR = 6.26 ± 4.30		RR = 9.66 ± 4.40		RR = 8.43 ± 3.99	
	RS = 10.3 ± 4.64		RS = 7.71 ± 4.04		RS = 9.20 ± 5.19		RS = 7.45 ± 4.05	
	SS = 6.44 ± 3.56		SS = 8.71 ± 4.69		SS = 8.1 ± 5.36		SS = 7.91 ± 4.76	

	*t‐*Test	*P* Value	*t‐*Test	*P* Value	*t‐*Test	*P* Value	*t‐*Test	*P* Value
RR *versus* RS	2.10	0.03*	1.93	0.05*	1.63	0.09	1.45	0.15
RR *versus* SS	6.95	6.43e^−10^*	2.41	0.01*	6.59	4.62e^−9*^	1.43	0.15

*Note*: RR, homozygote resistant; RS, heterozygote susceptible; SS, homozygote susceptible, *=p<0,05.

### 3.5 Effects of PPF‐based net Royal Guard on longevity

Survival curve of field female mosquitoes exposed to the treated net compared to untreated is shown in Fig. 2(A),(B). Overall for both field strains, Royal Guard induced a lower immediate mortality, coupled with a delayed effect many days post‐exposure.

The Tarone–Ware test used revealed that, wild *An. funestus* females in the control group lived longer (average life = 11.4 ± 0.4) than females in the Royal Guard (average life = 7.9 ± 0.2), (*χ* ^2^ = 6, *P* < 0.05); demonstrating that, despite the fact that this net has a reduced efficacy against resistant mosquitoes, there is a long‐term effect on *An. funestus* fitness. However, no significant difference was observed in the survival curve of field *An. gambiae* mosquitoes (*χ* ^2^ = 3.7, *P* = 0.05) in the control group (average lifespan = 9.9 ± 0.3) compared to the Royal Guard group (average lifespan = 8.7 ± 0.3). Comparison of survival curves for the two hybrid strains revealed a high immediate mortality following exposure to Royal Guard (Fig. 2(C),(D)), with significant variation observed over time.

The Tarone–Ware test revealed that FANG × FUMOZ unexposed females lived significantly longer (average life =12.8 ± 0.8) than those exposed to Royal Guard (average life = 8.2 ± 0.5), (*χ* ^2^ = 18.9, *P* < 0.001). Similar results were observed for KISUMU × MANGOUM females in the control group that lived significantly longer (average life = 8.7 ± 0.5) than those in the Royal Guard group (average life = 4.6 ± 0.3) (*χ* ^2^ = 43.1, *P* < 0.001). This result highlights the same negative impact of Royal Guard exposure on longevity in hybrid mosquitoes.

### 3.6 Impact of pyrethroid resistance markers on life traits parameters after Royal Guard exposure

#### 3.6.1 Impact of *An. gambiae* using the hybrid strain KISUMU × MANGOUM

##### 3.6.1.1 Impact of knockdown resistance with L1014F*‐Kdr‐W*

**Figure 3:**
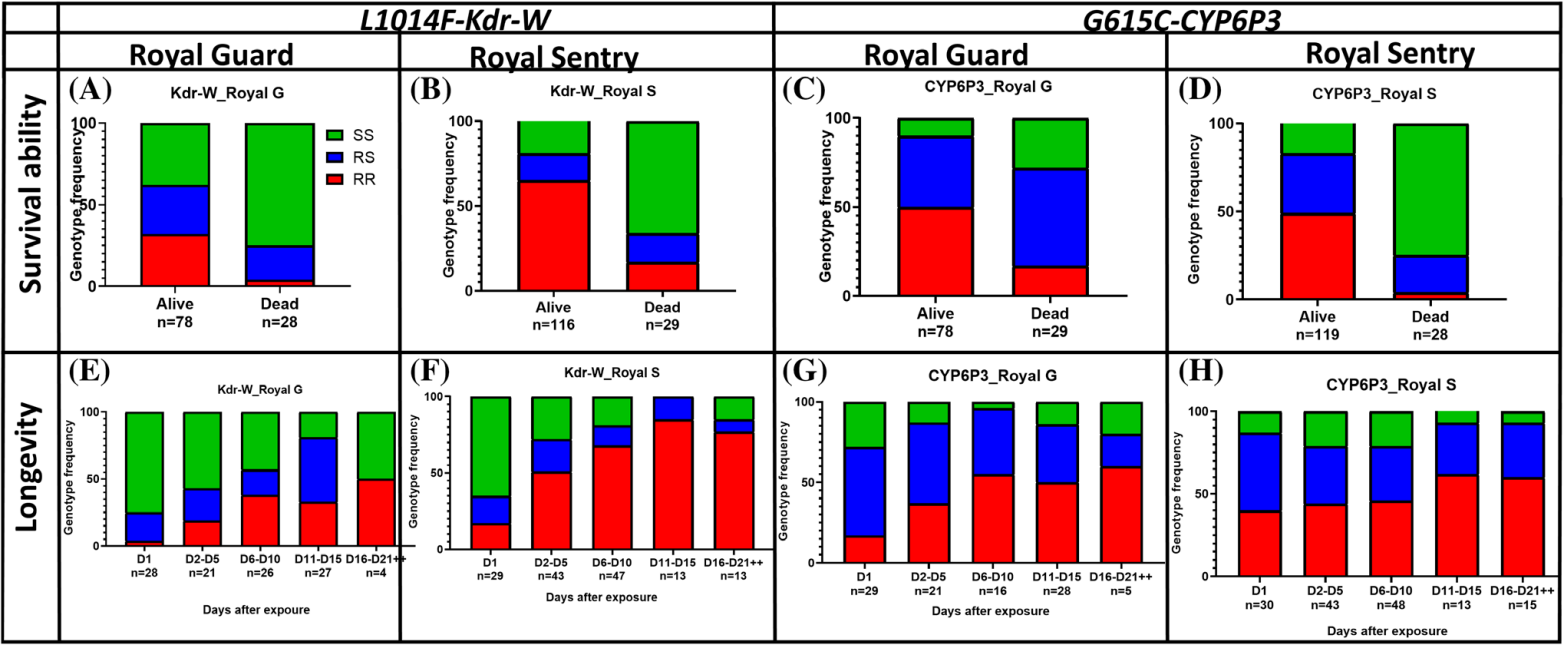
Correlation between L1014F_Kdr‐W and E205D‐CYP6P3 resistant markers and the efficacy of bed nets and life traits of KISUMU × MANGOUM. Genotype distribution of L1014F‐Kdr‐W mutation between alive and dead mosquitoes after exposure to Royal Guard (A), Royal Sentry (B) and G615C‐CYP6P3 between alive and dead after Royal Guard (C) and Royal Sentry (D) exposure. Genotype distribution of L1014F_Kdr‐W in surviving mosquitoes at different times post‐exposure to Royal Guard (E) and Royal Sentry (F) and G615C‐CYP6P3 for Royal Guard (G) and Royal Sentry (H). For genotype: RR, homozygote resistant; RS, heterozygote susceptible; SS, homozygote susceptible.

Genotyping of alive and dead mosquitoes post‐exposure to Royal Guard and Royal Sentry nets revealed a significant difference in the distribution of genotypes between both groups (*χ* ^2^ = 35.4; *P* < 0.0001 Fig. 3(A),(B)). The L1014F*‐Kdr‐W* homozygote‐resistant (RR) mosquitoes were significantly able to survive exposure to Royal Guard than homozygote‐susceptible (SS) mosquitoes [OR = 15.7; confidence interval (CI) = 5.3–43.2; *P* < 0.0001]. This correlation was even stronger at the allelic level, where the resistant allele had five times more chances to survive than those with the susceptible allele (OR = 5.4; CI = 2.7–10.9; *P* < 0.0001). Furthermore, survival curve analysis indicated that, mosquitoes with RR genotype survived for an average of 9.8 ± 4.6 days, whereas, heterozygote susceptible (RS) survived for about 8.07 ± 5.1 days and SS 4.8 ± 4.7 days (Fig. 3(E),(F) and Table S3). A similar pattern was observed for the Royal Sentry net, with RR genotype surviving for an average of 8.1 ± 5.2 days; RS about 5.9 ± 4.5 days; SS = 4.05 ± 4.4 days) post‐exposure (see later). No significant difference was observed in the distribution of genotypes for L1014F‐Kdr‐W (*χ* ^2^ = 1.08; *P* = 0.5) between blood fed females and unfed mosquitoes after exposure to all treated nets (Table S7).

##### 3.6.1.2 Impact of P450‐based metabolic resistance with E205D*‐CYP6P3*

Genotyping of the E205D*‐CYP6P3* marker revealed a significant difference in the distribution of genotypes between alive and dead mosquitoes (*χ* ^2^ = 27.1; *P* < 0.0001) as seen for the *kdr*. The E205D*‐CYP6P3* RR genotypes were significantly more able to survive exposure to Royal Guard than the SS mosquitoes (OR = 8.23; CI = 3.4–19.1; *P* < 0.0001) (Fig. 3(C)) and the RS mosquitoes (OR = 4.04; CI = 2.03–7.78; *P* < 0.0001). Similarly, a positive correlation was observed when comparing the alleles (Resistent *versus* Susceptible) (OR = 2.85; CI = 1.59–5.17; *P* = 0.0006). A similar profile was observed after exposure to Royal Sentry net, where RR were significantly more able to survive than SS (OR = 51.1; CI = 16.2–140.3; *P* < 0.0001) or RS (OR = 7.6; CI = 7. 6–2.5; *P* < 0.0002) (Fig. 3(D)). Assessing the impact of this CYP6P3 marker on mosquito longevity revealed a significantly higher proportion of RR genotypes from day 1 to day 21, while the frequency of SS genotypes decreased with time (Fig. 3(G),(H)). After exposure to Royal Gaurd, mosquitoes with RR‐CYP6P3 genotype had an average lifespan of 8.9 ± 5.2 days, RS = 6.02 ± 4.9 days; SS = 5.9 ± 6.5 whereas for Royal Sentry, the lifespan was 7.4 ± 5.4 days for RR, 6.2 ± 5.2 days for RS and 6 ± 4.6 days for SS (Fig. 6). For the blood‐feeding, no significant difference was observed in the distribution of genotypes between blood‐fed and unfed mosquitoes post‐exposure to Royal Guard (*χ* ^2^ = 2.1; *P* = 0.3) and Royal Sentry nets (*χ* ^2^ = 1.007; *P* = 0.6) although a higher proportion of RR (41%) was observed in those blood fed than unfed (33%) for Royal Guard (Table S7).

##### 3.6.1.3 Combined impact of target‐site and metabolic resistance L1014F*‐Kdr‐W/*E205D*‐CYP6P3*

The combined effect of the L1014F*‐Kdr* located across the sodium voltage dependent channel gene (Kdr; 2 L) and the *CYP6P3* cluster (CYP6P3; 2R) revealed an additive effect on life traits (Table S6). Double homozygote resistant (RR/RR) mosquitoes had significantly increased likelihood to survive exposure to Royal Guard (OR = 154; CI = 10.06–1734; *P* < 0.0001), and Royal Sentry net (OR = 16; CI = 3.02–81.8; *P* = 0.001) compared to their double homozygote susceptible counterparts (SS/SS) (Fig. S3). This suggests an additive effect of the two resistance mechanisms in reducing the efficacy of pyrethroid‐based nets. Over time, mosquitoes with RS/RS post‐exposure to Royal Guard had increased survival capacity compared to SS/SS (OR = 14.7; CI = 2.3–157.5; *P* = 0.0007). In contrast, no significant difference was observed for the ability to blood feed between RR/RR and SS/SS (OR = 1.8; CI = 0.3–8.4; *P* = 0.6).

#### 3.6.2 Impact in *An. funestus* using the hybrid FANG × FUMOZ

##### 3.6.2.1 Impact of P450‐based metabolic resistance with *CYP6P9a/b and 6.5Kb*

**Figure 4:**
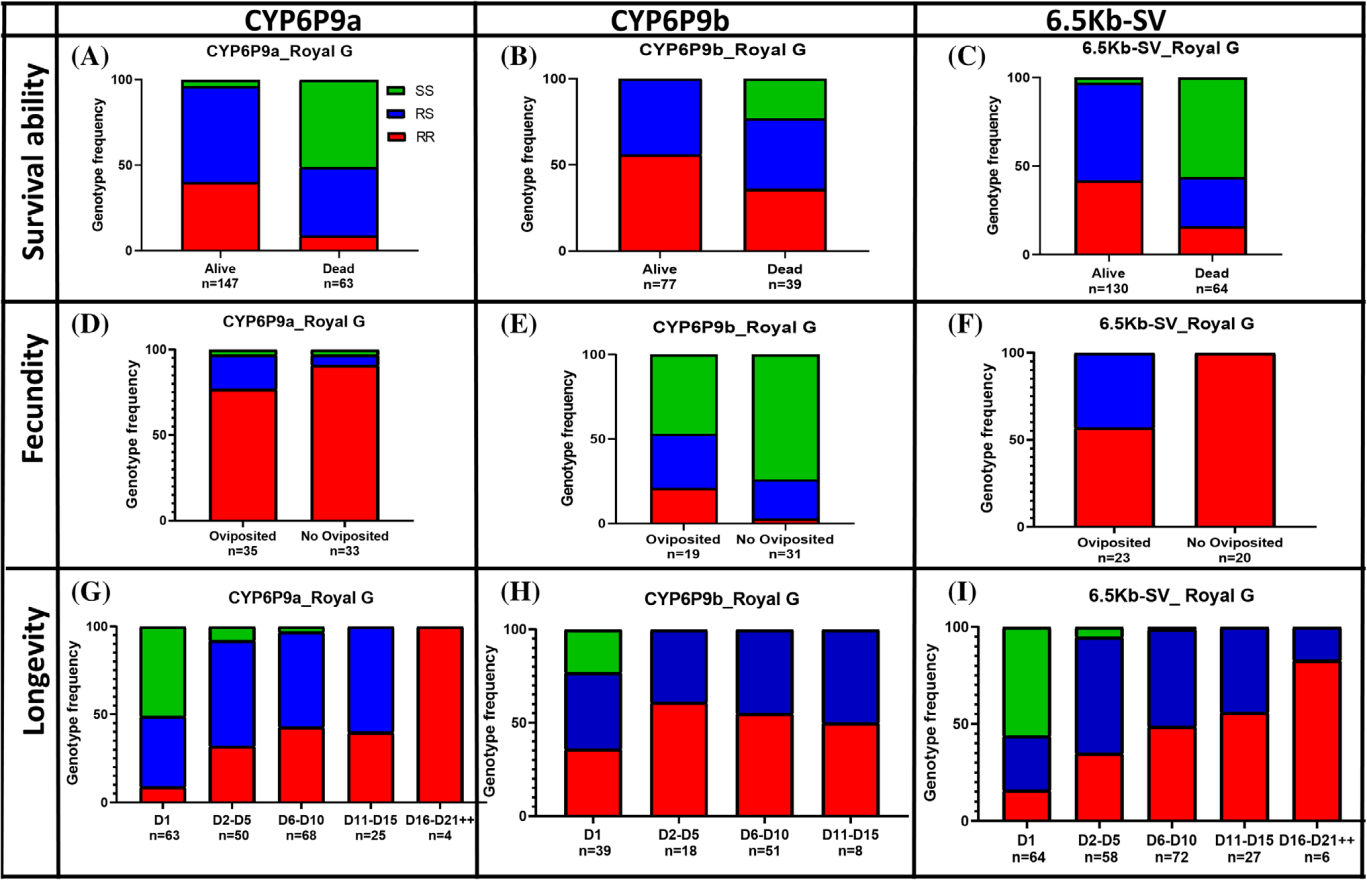
Correlation between CYP6P9a, CYP6P9b and 6.5Kb‐SV resistant markers and the efficacy of bed nets and life traits parameters of FANG × FUMOZ after Royal Guard exposure. Contingency graph showing the proportion of each genotype at the CYP6P9a locus in alive females compared to dead (A), the oviposited females compared to the non‐oviposited group (D), and the distribution of genotypes in surviving mosquitoes at different times post‐exposure to Royal Guard (G). Contingency graph showing the proportion of each genotype at the CYP6P9b locus in alive females compared to dead (B), the oviposited females compared to the non‐oviposited group (E), and the distribution of genotypes in surviving mosquitoes at different times post‐exposure to Royal Guard (H). Contingency graph showing the proportion of each genotype at the 6.5Kb‐SV locus in alive females compared to dead (C), the oviposited females compared to the non‐oviposited group (F), and the distribution of genotypes in surviving mosquitoes at different times post‐exposure to Royal Guard (I). For genotype: RR, homozygote resistant; RS, heterozygote susceptible; SS, homozygote susceptible.

As observed with *Kdr‐W* and *CYP6P3* previously, the distribution of the three P450 markers *CYP6P9a, CYP6P9b and 6.5Kb* was assessed in mosquitoes after exposure to Royal Guard (Fig. 4) and Royal Sentry nets (Fig. S2). Overall, the three markers showed the same trend for the different life parameters: longevity, blood feeding and fertility.

For *CYP6P9a*, a significant difference was observed in the distribution of genotypes between alive and dead mosquitoes for Royal Guard (*χ* ^2^ = 62.4; *P* < 0.0001). Evaluation of the OR showed that RR had a higher ability to survive exposure than RS (OR = 3.2; CI = 1.4–7.6; *P* = 0.005) and SS (OR = 56.7; CI = 15.9–164.9; *P* < 0.0001). A positive correlation at the allelic level was also found when comparing the alleles (Resistant *versus* Susceptible) (OR = 4.9; CI = 2.7–8.9; *P* < 0.0001). The impact of *CYP6P9a* marker on longevity revealed a difference in the distribution of genotypes over time (*χ* ^2^ = 314.2; *P* < 0.0001) (Fig. 4) from day 1 to day 21 and more (Table S3). Furthermore, mosquitoes with RR‐CYP6P9a genotype survived for an average of 7.5 ± 4.5 days, RS‐CYP6P9a = 5.5 ± 3.8 days; SS‐CYP6P9a = 1.5 ± 1.5 days (see later). For Royal Sentry, the lifespan was 15.6 ± 6.3 days for RR, 13.4 ± 7.6 days for RS and 1.31 ± 1.55 days for SS (see later).

For *CYP6P9b*, mosquitoes with RR genotype when exposed to Royal Guard net live an average of 5.9 ± 3.6 days, 5.3 ± 3.6 days for RS and 1 ± 0 day for SS whereas for Royal Sentry, this was 13.8 ± 6.05 days for RR, 14.2 ± 7.5 days for RS and 1.4 ± 1.6 days for SS. For the blood feeding assay, a significant difference was observed in the distribution of *CYP6P9a* genotypes between blood‐fed and unfed mosquitoes (*χ* ^2^ = 6.8; *P* = 0.03) (Table S7). Moreover, a significant difference was observed in the distribution of genotypes between the females that successfully laid eggs after blood meal and those that did not lay eggs (*χ* ^2^ = 9.5; *P* = 0.0008) (Fig. 4(D)) as RR mosquitoes have a greater ability to lay eggs than RS (OR = 2.7; CI = 1.4–4.8; *P* = 0.0003) after Royal Guard exposure. Females with the CYP6P9a_RR genotype tended to lay more eggs (56. 04 ± 3.7) than CYP6P9a_RS (46.5 ± 10.9) (*χ* ^2^ = 9.3; *P* = 0.0002).

For the *6.5Kb‐SV* allele, similar observations were also found for the fecundity ability showing that RR females were more likely to lay eggs than RS females (OR = 75.4; CI = 12.49–774.8; *P* < 0.0001) following exposure to Royal Guard (Fig. 4(C)). It was also noted that females with the 6.5Kb‐SV_RR genotype laid more eggs (54.8 ± 10.9) than those with the 6.5Kb‐SV_RS genotype (53.5 ± 5.9) after Royal Guard exposure (*χ* ^2^ = 0.3; *P* = 0.5).

##### 3.6.2.2 Combined impact of multiple P450 resistance alleles

The impact of various combinations of genotypes between *CYP6P9a, CYP6P9b and 6.5Kb‐SV*; on the efficacy of LLINs revealed that triple homozygote resistant (RR/RR/RR) had significantly greater chance to survive Royal Guard exposure than SS/SS/SS (OR = 130; CI = 28.55–460.7; *P* < 0.0001) (Fig. S4). A similar observation was obtained for CYP6P9a_RR/CYP6P9b_RR compared to SS/SS (OR = 880; CI = 49.87–9194; *P* < 0.0001) (Table S6). Post exposure to Royal Guard, RR/RR had the greatest ability to survive and live long compared to SS/SS (OR = 42.8; CI = 6.15–507; *P* < 0.0001) (Fig. S5). Mosquitoes with RR/RR genotypes for *CYP6P9a* and *6.5Kb‐SV* had more chance to take a blood meal than RS/RS (OR = 46.5; CI = 7.20–491.3; *P* < 0.0001). RR/RR mosquitoes also had an increased ability to lay eggs compared to RS/RS mosquitoes (OR = 6.55; CI = 2.46–0.40; *P* < 0.0001) (Fig. S5).

#### 3.6.3 Long‐term impact of exposure with field populations of *An. funestus* using the G454A‐CYP9K1 and L119F‐GSTe2 alleles

##### 3.6.3.1 Impact of the P450 G454A‐CYP9K1 allele

**Figure 5:**
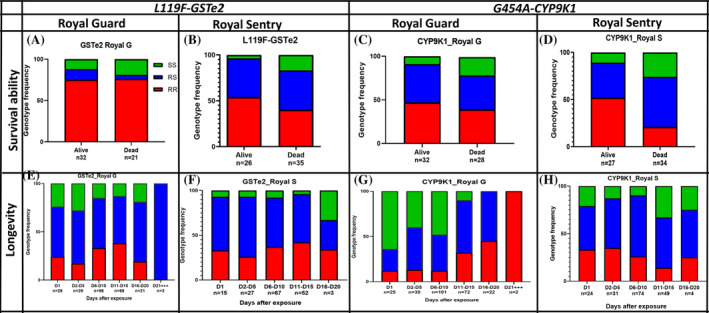
Influence of the L119F_GSTe2 and G454A‐CYP9K1 mutations on the efficacy of bed nets and longevity of field *Anopheles funestus* from Elendé. Genotype distribution of L119F_GSTe2 mutation between alive and dead mosquitoes after exposure to Royal Guard (A) and Royal Sentry (B) and G454C_CYP9K1 mutation for Royal Guard (C) and Royal Sentry (D). Long‐term impact of L119F_GSTe2 at different time points after Royal Guard exposure (E) and Royal Sentry (F) and G454A_CYP9K1 for Royal Guard exposure (G) and Royal Sentry (H). For genotype, RR, homozygote resistant; RS, heterozygote susceptible; SS, homozygote susceptible.

**Figure 6:**
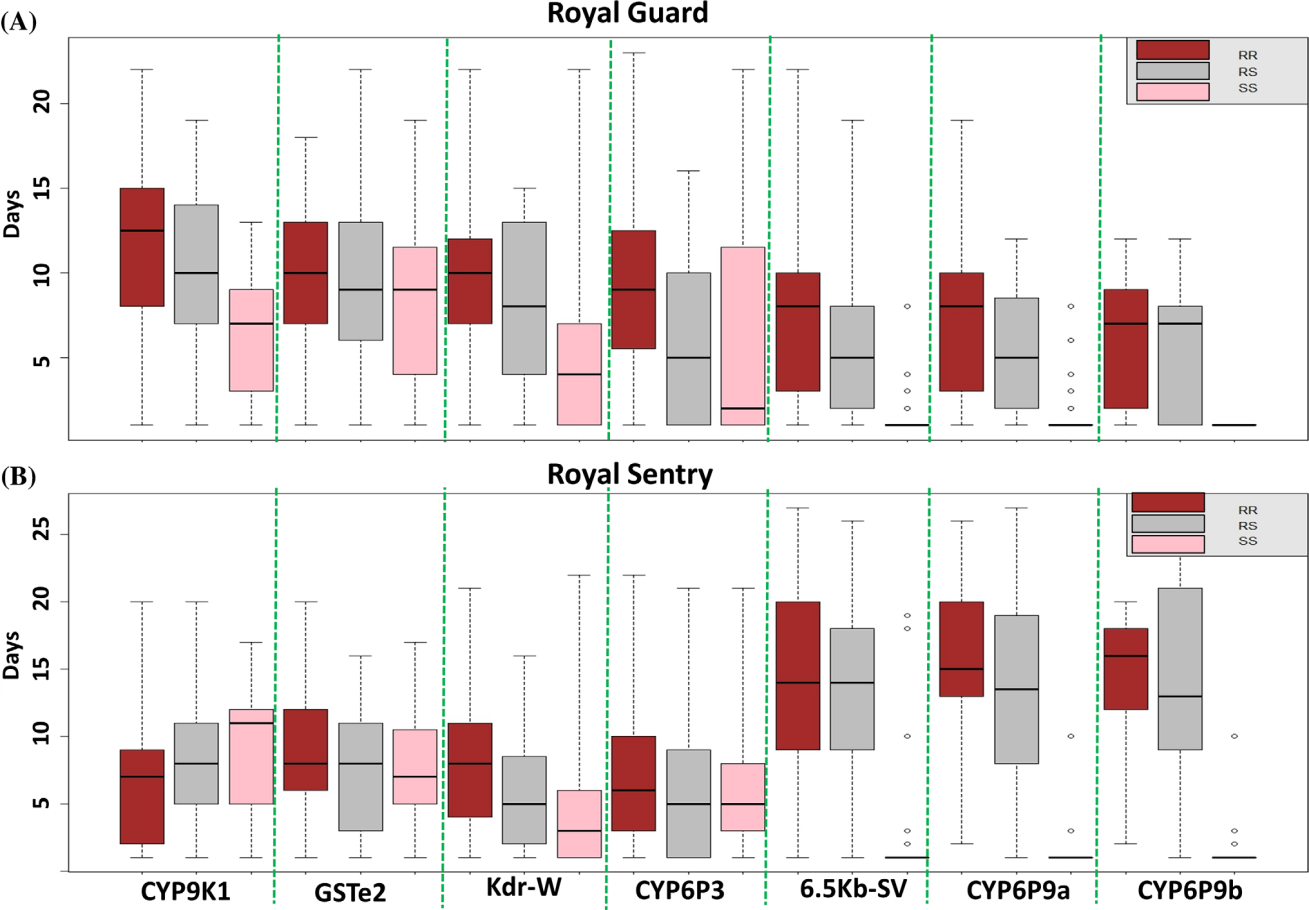
Distribution of mean survival day for each genotype after exposure to Royal Guard and Royal Sentry. (A) The box plot for the genotype distribution for Royal Guard and (B) for Royal Sentry. For genotype: RR, homozygote resistant; RS, heterozygote susceptible; SS, homozygote susceptible.

Genotyping revealed that *CYP9K1* is responsible for reducing bed net efficacy for both Royal Guard and Royal Sentry nets regardless of physical status (washed/unwashed) or insecticide concentration on the net. Genotyping of alive and dead mosquitoes post‐exposure to Royal Guard net revealed that *G454A‐CYP9K1* RR mosquitoes were significantly more able to survive exposure to Royal Guard than SS mosquitoes (OR = 2.8; CI = 1.19–6.5; *P* = 0.03) (Fig. 5(C)). The comparison of genotype frequencies showed a decrease in the proportion of CYP9K1_SS from day 1 to day 12 while the CYP9K1_RR increased from day 1 to day 20 or more showing that, resistant mosquitoes have more chance to survive than susceptible ones (Fig. 5(G)). Assessing the OR revealed that mosquitoes with the resistant allele lived longer compared to those with the susceptible allele (OR = 7.5; CI 95%: 1.04–21.3; *P* < 0.001) (Table S4). In addition, mosquitoes with RR‐CYP9K1 genotype live an average of 12 ± 5.1 days, RS‐CYP9K1 = 10.3 ± 4.6 days; SS‐CYP9K1 = 6.4 ± 3.5 days (Fig. 6).

##### 3.6.3.2 Impact of L119F*‐GSTe2*

Genotyping of L119F*‐GSTe2* revealed no significant difference in the distribution of genotypes between alive and dead mosquitoes (*χ* ^2^ = 5.1; *P* = 0.07) (Fig. 5(A)). However, RS mosquitoes were significantly able to survive exposure to Royal Guard than SS mosquitoes (OR = 4.1; CI = 1.1–13.6; *P* = 0.03). The findings revealed a reduction of SS mosquitoes from day 1 to day 21 after Royal Guard exposure (Fig. 5(E)). Contrarily, the RR genotype exhibited a significantly higher likelihood of survival compared to RS (OR = 46.1; CI = 7.9–481.7; *P* < 0.0001). However, no significant difference was observed in the allele distribution over time (Table S4). For Royal Guard, mosquitoes with RR‐GSTe2 genotype live an average of 9.7 ± 4.4 days, 9.2 ± 5.2 days for RS and 8.1 ± 5.3 days for SS whereas for Royal Sentry net, this was 8.4 ± 3.9 days for RR, 7.4 ± 4.05 days for RS and 7.9 ± 4.7 days for SS (Fig. 6).

##### 3.6.3.3 Combined impact of G454A‐CYP9K1 and L119F‐GSTe2

Analysis of the combined impact of *G454A‐CYP9K1* and *L119F‐GSTe2* pyrethroid‐resistant markers revealed that, both markers when combined allow the vectors to survive exposure and live longer (Fig. S6). Mosquitoes with RR/RR genotype had significantly greater survival than SS/SS (OR = 100.8; CI = 16.4–1062; *P* < 0.0001). The same trend was observed for the long‐term effect where, RR/RR mosquitoes have more chance to live longer than SS/SS (OR = 60; CI = 3.1–732.5; *P* = 0.002) (Table S5).

## 4 DISCUSSION

This study has extensively assessed the efficacy of Royal Guard and the post‐exposure effect on different life trait parameters of malaria vectors, using DNA markers for metabolic and knockdown resistance. It has been revealed that despite the fact that pyrethroid‐resistant mosquitoes have a higher ability to survive and live longer after exposure to Royal Guard net, this net significantly affects their lifespan, and their blood feeding ability, reducing their fecundity and fertility.

### 4.1 Pyriproxyfen‐based net Royal Guard displayed higher efficacy on field *An. funestus* and *An. gambiae* compared to the standard net Royal Sentry

Higher efficacy of the dual AI net Royal Guard (mortality for unwashed and washed) was observed compared to the pyrethroid‐only net Royal Sentry, using hybrid mosquitoes. The low mortality response of the field population is common to other pyrethroid nets evaluated in Cameroon^21^, ^37^, ^38^ and is partly attributed to insecticide resistance. Malaria vectors have developed a high level of resistance to pyrethroids through metabolic and target site resistance across the country, leading to the loss of efficacy of bed nets as previously reported.^37^, ^39^, ^40^, ^41^ Despite a high level of resistance, a significant difference was observed between Royal Guard unwashed and 20‐times washed (70% mortality for 0 washes and 18% over 20 washes), the same was observed for Royal Sentry net (58% mortality for 0 washes and 26% over 20 washes), indicating a significant loss of efficacy after multiple washes. Similar observations were made in Côte d'Ivoire^42^ with washed and unwashed Olyset Duo. The higher mortality found with Royal Guard nets compared to the pyrethroid‐only net Royal Sentry could be attributed to the higher alpha‐cypermethrin (208 mg/m^2^) concentration compared to Royal Sentry (alpha‐cypermethrin 203 mg/m^2^), as previously proposed.^16^ In addition, as both pyrethroids and PPF have been shown to be metabolized by the same cytochrome P450s genes, this increased mortality observed in the PPF + alpha‐cypermethrin combination may be indicative of a saturation effect, whereby mosquitoes are unable to simultaneously detoxify the two active ingredients on the net.^43^ These findings are supported by those from experimental hut trials in Benin and Côte d'Ivoire, where *Anopheles* are highly resistant to pyrethroids, which also found higher levels of mortality with AI nets than LLINs.^42^, ^44^

### 4.2 Exposure to the pyriproxyfen‐based net Royal Guard induced sterility in mosquitoes while also affecting blood feeding and longevity

This study revealed that Royal Guard induced significantly higher blood‐feeding inhibition than untreated net, highlighting the negative effect of exposure on blood meal intake. Previous studies have already reported similar observations in semi‐field conditions^16^, ^42^, ^45^ and in the laboratory.^46^, ^47^ However, Harris *et al*. demonstrated that, the timing of blood feeding in relation to PPF exposure significantly affects mosquito offspring production.^48^ The results presented in this study clearly show that Royal Guard 20W had the highest reductions in oviposition compared to Royal Sentry 20W, resulting in a significant reduction in fecundity and in offspring compared to the untreated group. However, the proportion of reduction in fecundity and in offspring did not differ significantly between the two treated nets, which could be explained by the potential errors in the counting process, as suggested in the literature,^49^, ^50^ where they clearly demonstrated that ovary dissection is a more sensitive and specific tool for measuring sterility than the oviposition method. That is why we subsequently used the ovary dissection method after testing the bed net in cone test. Thus, a higher infertility rate was observed in the group exposed to Royal Guard 20W compared to the Royal Sentry 20W or control group, suggesting that, despite multiple washes, the nets still had enough PPF to sterilize some mosquitoes. A fine scale observation of the egg development showed that 4 days after the blood feeding, eggs in the ovaries of the females not exposed to PPF underwent successive developmental stages known as Christopher stages until stage V with visible lateral floats. In contrast, females exposed to PPF‐treated nets exhibited significantly delayed egg development. Their follicles never reached full maturity and displayed various stages of disruption. Some eggs remained in the early round stage (Christopher stage II), while others appeared elongated and dark (Christopher stage IV), indicating potential egg abortion. These findings are consistent with previous studies.^6^, ^48^ To validate this sterilizing effect of PFF, we performed CDC bottle assay with insecticide alone (acetone, alpha‐cypermethrin and PFF). The results showed a very high infertility rate in susceptible strain KISUMU (100%), *An. funestus* (74.54%) and *An. gambiae* (96%). Similarly, another study using *Anopheles arabiensis* found that eggs were 100% abnormal after being exposed to PPF.^48^

The differential expression between PPF‐treated and control mosquitoes was assessed by qRT‐PCR. Our study revealed that, 24 h after blood meal, the aberrant expression patterns of reproductive genes in PPF‐treated mosquitoes undoubtedly contributed to the disrupted egg maturation. In a normal case, the first gonotrophic cycle can be divided into two steps separated by blood feeding: the previtellogenic phase (before a blood meal) and the vitellogenic phase (post‐blood meal).^51^ During the post‐emergence development in mosquitoes, a JH‐regulated transcriptional factor induces expression of Kr‐h1 before and after the blood feeding, driving sequential waves of gene expression and prompting the growth of the primary follicle.^52^, ^53^ The follicles then enter an arrested stage of development, awaiting a blood meal. Then, 24 h after the blood meal, 20E synthesis begins, leading to a rapid decrease in JH and Kr‐h1 levels.^54^ At approximately 30 h post‐blood meal, 20E levels drop back to basal levels, signaling the end of vitellogenin synthesis. After 72 h, the egg maturation is complete and female mosquitoes are ready for oviposition.^55^ In contrast, this two‐stage hormonal process is disrupted in the presence of PPF, which blocks the complete formation of eggs in the ovaries, enhancing the sterilizing effect. Ahmed *et al*. using *Aedes* mosquitoes, showed that PPF considerably enhanced the expression of the cascade of reproductive genes (EcRA, E75A, HR3) mediated by 20E, which should be at low basal levels before the blood meal.^52^ After a blood meal, the PPF analogue to JH had the opposite effect, inducing the expression of Kr‐h1 keeping its expression high at a time when it would normally be low, and reducing the expression of the 20E.^52^, ^53^ In the present study, the expression level was assessed only 24 h after the blood meal, showing the increased expression of Kr‐h1 in the PFF group.

In this study, PPF exposure significantly reduced the lifespan of mosquitoes in both hybrids and field strains. A similar life‐shortening effect was observed after exposure to pyrethroid‐only nets, which have previously been shown to reduce adult longevity^18^, ^41^ and also to the mixture of permethrin + PPF.^56^ Equally, exposure to Royal Guard had a strong negative effect on longevity higher than the Royal Sentry net, suggesting that the reduction in lifespan is primarily due to the combination of two active ingredients.^47^ To transmit malaria parasites, mosquitoes need to survive for 10–14 days for the parasite to develop between their first and subsequent blood meals. The shorter lifespan observed in the laboratory could also occur in the field; consequently, PPF‐based nets would likely reduce malaria transmission by lowering the number of infected mosquitoes. This idea is supported by a decrease in parous mosquitoes seen in villages with PPF‐treated nets, as shown in a study in Burkina Faso^57^ and highlights the entomological impact of interventions such as PPF‐treated nets in combating malaria. However, the lack of significant epidemiological protective effect observed in the randomized‐controlled trial in Benin^17^ suggests that their effectiveness could be impacted by other factors including insecticide resistance.

### 4.3 Pyrethroid‐resistant markers confer a greater chance of survival after exposure to Royal Guard and impact life traits

Genotyping of various metabolic markers of detoxification genes (*CYP6P9a*, *CYP69b*, *6.5Kb‐SV*, *CYP9K1*, *CYP6P3* and *GSTe2*) and target site gene (*Kdr*) revealed that these resistance markers are reducing the efficacy of bed nets as previously reported.^24^, ^32^, ^33^, ^58^, ^59^, ^60^ Moreover, an additive effect of these markers was observed as a greater reduction of bed net efficacy was observed when markers were combined, for example triple or double homozygote resistant mosquitoes for (*CYP6P9a/CYP99b/6.5Kb‐SV*), (*CYP9K1/GSTe2*) and (*Kdr/CYP6P3*) were more able to survive exposure to pyrethroid‐only and PPF‐based nets compared to other genotypes.

Females possessing the resistant allele for each resistant marker exhibited increased longevity, potentially contributing to an increase in vectorial capacity of resistant mosquitoes. This is because the extrinsic incubation period of *Plasmodium* parasites is more likely to be completed, allowing females to further take blood meals with the infective sporozoite stage. Similar observations on resistant alleles selected after exposure were found for *CYP6P9a* and *GSTe2*.^19^, ^41^ Nevertheless, other studies have shown that in absence of selection pressure, there is a fitness cost to resistant mosquitoes explaining why the longevity of the female *An. funestus* was not impacted by the resistant allele.^28^ In fact, P450 monooxygenases and glutathione‐S‐transferases (GSTs) are known to significantly alter the levels of reactive oxygen species in insects.^61^ GSTs protect mosquitoes from oxidative stress, leading to increased longevity, whereas overproduction of P450 monooxygenases results in increased oxidative stress and thus reduced insect longevity.^62^

Our study is the first to demonstrate an association between the newly identified P450‐based resistance marker (E205D) of the *An. gambiae CYP6P3* gene and the Knockdown resistance (*Kdr*) towards post‐exposure survival. These results suggest that resistant alleles for these markers not only enhance the ability to survive immediate exposure to pesticides but may also help mosquitoes overcome the deleterious effects of the insecticide several days post‐exposure. A similar decrease in mortality has been observed in *Aedes aegypti* harboring the *kdr* allele, although this was associated with decreased longevity.^63^ Likewise, a reduction in longevity has been reported for the Rdl dieldrin resistance marker in some strains of malaria vectors *An. gambiae* and *Anopheles stephensi*.^64^ Furthermore, the observed resistant pattern is attributable to alpha‐cypermethrin impregnated bed nets, as resistance to PPF formulations has not yet been reported in mosquitoes,^65^ and no cross‐resistance between PPF and other public health insecticides has been observed.

Although resistance mechanisms enable mosquitoes to survive and live longer under continuous insecticide pressure, these adaptations are costly and can negatively impact the fitness of mosquitoes, including body size, larval development time, fecundity, fertility, mating competitiveness and blood feeding capability.^64^, ^66^ In fact, in this study, resistant mosquitoes (RR) exhibited a greater ability to lay eggs than RS and SS. This finding aligns with previous research by Tchouakui *et al*. that demonstrated that field‐resistant mosquitoes exhibited reduced fecundity, slower larval development but increased adult longevity.^67^ Conversely, a DNA‐based assay designed for cytochrome P450‐mediated resistance (*CYP6P9a*‐R) in *An*. *funestus* showed that mosquitoes carrying this P450‐resistant allele survived and succeeded in blood feeding more often than did susceptible mosquitoes when exposed to Royal Guard; which was already demonstrated for insecticide‐treated net PermaNet 2.0.^24^ Other studies suggested that *CYP6P9a* marker is linked with the feeding success and blood meal size of *An*. *funestus*. However, no correlation was found between the expression of *CYP6P9a* and that of genes encoding for salivary proteins involved in the blood meal process.^68^ So, P450‐based metabolic and target site resistance may influence the longevity, blood feeding process and fecundity ability of resistant mosquitoes against PPF‐based nets, consequently impacting their ability to transmit malaria parasites. This calls for the development of other management strategies as previously reported.^69^

## 5 CONCLUSION

This study investigated the efficacy and long‐term impact of exposure to the dual AI‐net Royal Guard on malaria vectors, revealing that the combination of PPF and pyrethroids on bed nets has the potential to provide better protection beyond the immediate mortality observed. However, the reduction of efficacy observed in pyrethroid‐resistant mosquitoes is a major concern for the sustainability of pyrethroid‐based interventions. Thus, results in this study encourage future strategies using new insecticides with different modes of action than pyrethroids to reduce the selection pressure, allowing the rotation strategy to be effectively implemented.

**ABBREVIATIONS**
0W: Unwashed nets
20E: 20‐hydroxyecdysone
20W: 20 Times‐washed nets
AI: Active ingredients
CI: Confidence interval
EcRA: Ecdyson receptor isoform A
HR3: Hormone receptor 3
Kdr: Knockdown resistance
Kr‐h1: Kruppel‐homologue 1
Met: Methoprene tolerant
OR: Odd ratio
RR: homozygote resistant
RS: heterozygote susceptible
SS: homozygote susceptible

## FUNDING INFORMATION

This work was supported by a Wellcome Trust Senior Research Fellowships in Biomedical Sciences to CSW (217188/Z/19/Z) and a Bill and Melinda Gates Foundation grant to CSW (INV‐006003). The funders had no role in study design, data collection and analysis, decision to publish, or preparation of the article.

## CONFLICT OF INTEREST STATEMENT

The authors declare no competing interests.

## AUTHOR CONTRIBUTIONS

Conceptualization: CSW. Design: MT and CSW. Data curation: ESN‐Y and MT. Formal analysis: ESN‐Y and MT. Funding acquisition: CSW. Investigation: ESN‐Y, MT, RFT, DF and VBN‐F. Methodology: ESN‐Y, MT and CSW. Validation: MT and CSW. Writing – original draft: ESN‐Y. Writing – review and editing: ESN‐Y, MT and CSW. All authors read and approved the final article.

## CONSENT FOR PUBLICATION

Not applicable.

## Supporting information


**Figure S1.** Impact of Royal Guard exposure on blood‐feeding after tunnel assay and dissection of ovaries from female mosquitoes. Pictures of ovaries from females in the control group (A) with fertile eggs having lateral floats (Stage V) and ovaries from females in Royal Guard group with Eggs infertile or non‐fecund with a round shape and lacking floats (Stages IIb) (B) and (Stage IV) (C). Blood feeding inhibition after tunnel test (D); dissection of ovaries from adult female mosquitoes was done 24 h after the blood feeding. Note that (A), (B), and (C) were photographed at 400× magnification. Asterisk (*) represents the level of significance for each LLIN relative to the control net and between the two nets, ns: non‐significant difference compared to the control or between the two nets, 0W: unwashed nets, 20W: 20 times‐washed nets.
**Figure S2.** Correlation between CYP6P9a, CYP6P9b and 6.5Kb‐SV resistant markers and the efficacy of Royal Sentry and life traits parameters of FANG × FUMOZ. Contingency graph showing the proportion of each genotype at the CYP6P9a locus in alive females compared to dead (A), the oviposited females compared to the non‐oviposited group (D), the distribution of genotypes for the oviposited females (G) and the distribution of genotypes in surviving mosquitoes at different times post‐exposure to Royal Sentry (J). Contingency graph showing the proportion of each genotype at the CYP6P9b locus in alive females compared to dead (B), the oviposited females compared to the non‐oviposited group (E), the distribution of genotypes for the oviposited females (H) and the distribution of genotypes in surviving mosquitoes at different times post‐exposure to Royal Sentry (K). Contingency graph showing the proportion of each genotype at the 6.5Kb‐SV locus in alive females compared to dead (C), the oviposited females compared to the non‐oviposited group (F), the distribution of genotypes for the oviposited females (I) and the distribution of genotypes in surviving mosquitoes at different times post‐exposure to Royal Sentry (M). For genotype: RR, homozygote resistant; RS, heterozygote susceptible; SS, homozygote susceptible.
**Figure S3.** Correlation between Kdr‐W, CYP6P3, CYP6P9a, CYP6P9b and 6.5Kb‐SV resistant markers and the blood feeding ability Royal Guard and Royal Sentry exposure. (A, B) The combined effect of the correlation between the L1014F_Kdr‐W/G615C_CYP6P3 genotypes and ability to survive to Royal Guard and (C, E) the cumulative effect of the longevity for Royal Guard and (D, F) to Royal Sentry.
**Figure S4.** Cumulative effect of L1014F_Kdr‐W/E205D_CYP6P3 on life traits parameters after exposure to bed nets. (A) The combined effect of the three mechanisms CYP6P9a/CYP6P9b/6,5Kb‐SV for Royal Guard, (E) for Royal Sentry; (B, F) the combined effect of two mechanisms CYP6P9a/6.5Kb‐SV for Royal Guard and Royal Sentry; (C, G) the combined effect of two mechanisms CYP6P9b/6.5Kb‐SV for Royal Guard and Royal Sentry; (D, H) the combined effect of two mechanisms CYP6P9a/CYP6P9b for Royal Guard and Royal Sentry.
**Figure S5.** Cumulative effect of CYP6P9a, CYP6P9b, and 6,5Kb‐SV on life traits parameters after exposure to bed nets. (A, D) The combined effect of two mechanisms CYP6P9a/6.5Kb‐SV for Royal Guard, (G, J) for Royal Sentry on fecundity and longevity; (B, E) the combined effect of two mechanisms CYP6P9b/6.5Kb‐SV for Royal Guard, (H, K) for Royal Sentry on fecundity and longevity; (C, F) the combined effect of two mechanisms CYP6P9a/CYP6P9b for Royal Guard, (I, M) for Royal Sentry on fecundity and longevity.
**Figure S6.** Cumulative effect of L119F_GSTe2/G454A_CYP9K1 on life traits parameters after exposure to bed nets. (A, B) The combined effect of the correlation between the L119F_GSTe2/G454A_CYP9K1 genotypes on longevity after Royal Guard and Royal Sentry exposure at the genotypic level; (C, D) at the allelic level.

**Table S1.** List of primers used in quantitative real‐time PCR.
**Table S2.** List of primers and probes used for the detection of known resistance markers.
**Table S3.** Evaluation of the association between different genotypes of the Kdr‐W, CYP6P3, CYP6P9a, CYP6P9b and 6.5Kb‐SV mutations and female longevity in exposed mosquitoes.
**Table S4.** Evaluation of the association between different genotypes of GSTe2 and CYP9K1 mutation and female longevity in exposed mosquitoes.
**Table S5.** Evaluation of the association between combined genotypes of GSTe2/CYP9K1 mutation and female longevity in exposed mosquitoes.
**Table S6.** Evaluation of the association between combined genotypes of Kdr/CYP6P3 and CYP6P9a/CYP6P9b mutation and female longevity in exposed mosquitoes.
**Table S7.** Correlation between Kdr‐W, CYP6P3, CYP6P9a, CYP6P9b and 6.5Kb‐SV resistant markers and the blood feeding ability of Royal Guard and Royal Sentry exposure.

## Data Availability

The data that supports the findings of this study are available in the supplementary material of this article.
