# Supplementary material for: Long‐term impact of exposure to Royal Guard, a pyriproxyfen‐based bed net, on pyrethroid‐resistant malaria vectors from Cameroon using DNA‐based metabolic resistance markers
Source: Pest Manag Sci. 2025 Jan 23;81(4):2165–80. doi: 10.1002/ps.8615 (PMC11906912; doi:10.1002/ps.8615)
Supplement: Supplementary file 1 — Figure S1. Impact of Royal Guard exposure on blood‐feeding after tunnel assay and dissection of ovaries from female mosquitoes. Pictures of ovaries from females in the control group (A) with fertile eggs having lateral floats (Stage V) and ovaries from females in Royal Guard group with Eggs infertile or non‐fecund with a round shape and lacking floats (Stages IIb) (B) and (Stage IV) (C). Blood feeding inhibition after tunnel test (D); dissection of ovaries from adult female mosquitoes was done 24 h after the blood feeding. Note that (A), (B), and (C) were photographed at 400× magnification. Asterisk (*) represents the level of significance for each LLIN relative to the control net and between the two nets, ns: non‐significant difference compared to the control or between the two nets, 0W: unwashed nets, 20W: 20 times‐washed nets. Figure S2. Correlation between CYP6P9a, CYP6P9b and 6.5Kb‐SV resistant markers and the efficacy of Royal Sentry and life traits parameters of FANG × FUMOZ. Contingency graph showing the proportion of each genotype at the CYP6P9a locus in alive females compared to dead (A), the oviposited females compared to the non‐oviposited group (D), the distribution of genotypes for the oviposited females (G) and the distribution of genotypes in surviving mosquitoes at different times post‐exposure to Royal Sentry (J). Contingency graph showing the proportion of each genotype at the CYP6P9b locus in alive females compared to dead (B), the oviposited females compared to the non‐oviposited group (E), the distribution of genotypes for the oviposited females (H) and the distribution of genotypes in surviving mosquitoes at different times post‐exposure to Royal Sentry (K). Contingency graph showing the proportion of each genotype at the 6.5Kb‐SV locus in alive females compared to dead (C), the oviposited females compared to the non‐oviposited group (F), the distribution of genotypes for the oviposited females (I) and the distribution of genotypes in survivin [file PS-81-2165-s001.docx]

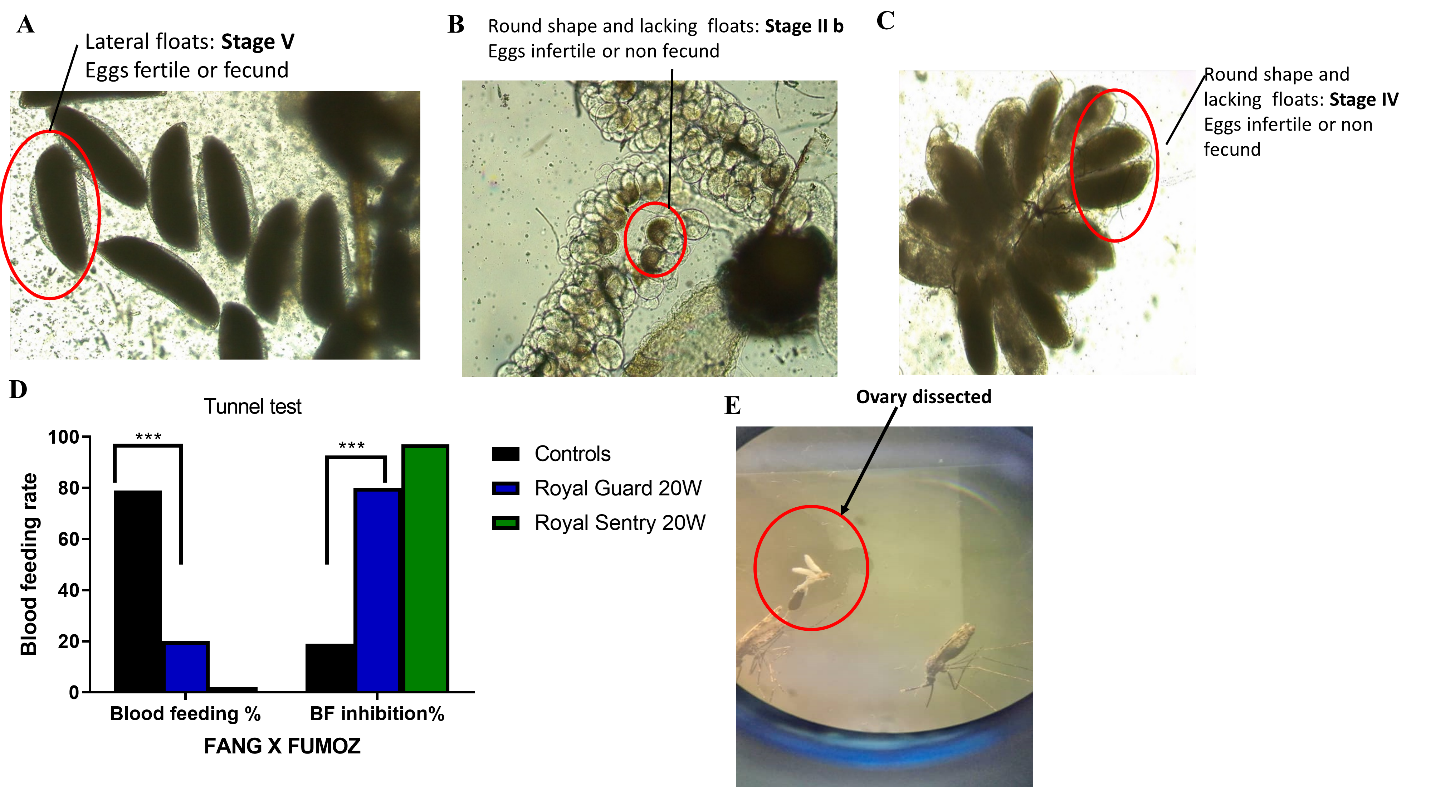


**Figure S1:** Impact of RG exposure on blood-feeding after tunnel assay and dissection of ovaries from female mosquitoes. Pictures of ovaries females from Control group (A) with eggs fertile having lateral floats (Stage V) and ovaries from females in Royal G group with Eggs infertile or non fecund with round shape and lacking floats (Stages IIb) (B) and (Stage IV) (C). Blood feeding inhibition after tunnel test (D); Dissection of ovaries from adult female mosquitoes were done 24 h after the blood feeding. Note that A, B, and C were shot at 400x magnification. *represents the level of significance for each LLIN relative to the control net and between the two nets, ns: non significate difference compare to the control or between the 2 nets, 0w: unwashed nets, 20w: 20 times-washed nets.


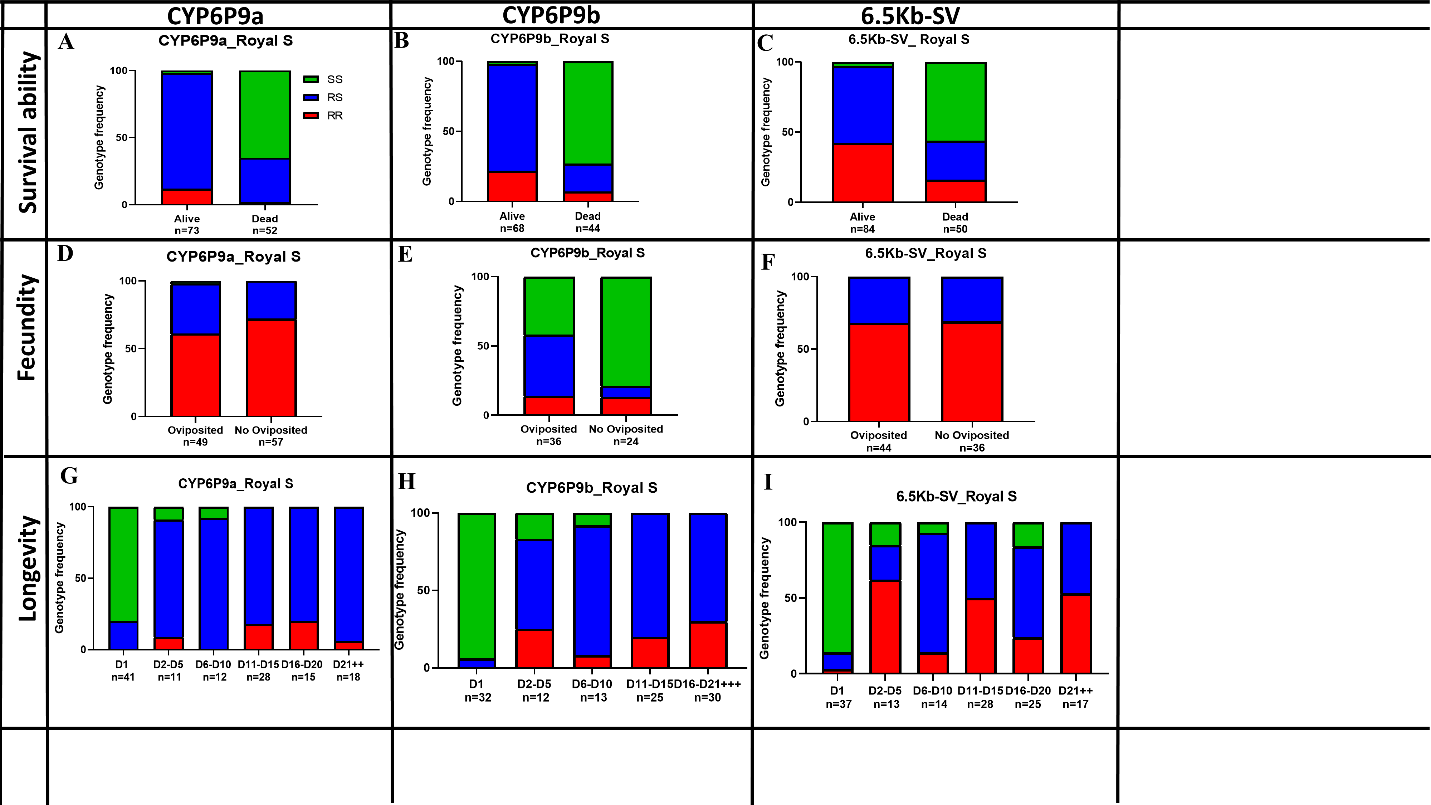


**Figure S2**: Correlation between CYP6P9a, CYP6P9b and 6.5Kb-SV resistant marker and the efficacy of RS and life traits parameters of FANG X FUMOZ. Contingency graph showing the proportion of each genotype at the CYP6P9a locus in alive females compared to dead (A), the oviposited females compared to the non-oviposited group (D), and the distribution of genotypes in surviving mosquitoes at different times post‑exposure to Royal S (G). Contingency graph showing the proportion of each genotype at the CYP6P9b locus in alive females compared to dead (B), the oviposited females compared to the non-oviposited group (E), and the distribution of genotypes in surviving mosquitoes at different times post‑exposure to Royal S (H). Contingency graph showing the proportion of each genotype at the 6.5Kb-SV locus in alive females compared to dead (C), the oviposited females compared to the non-oviposited group (F), and the distribution of genotypes in surviving mosquitoes at different times post‑exposure to Royal S (I). For genotype, RR: homozygote resistant, RS: heterozygote, SS: homozygote susceptible.


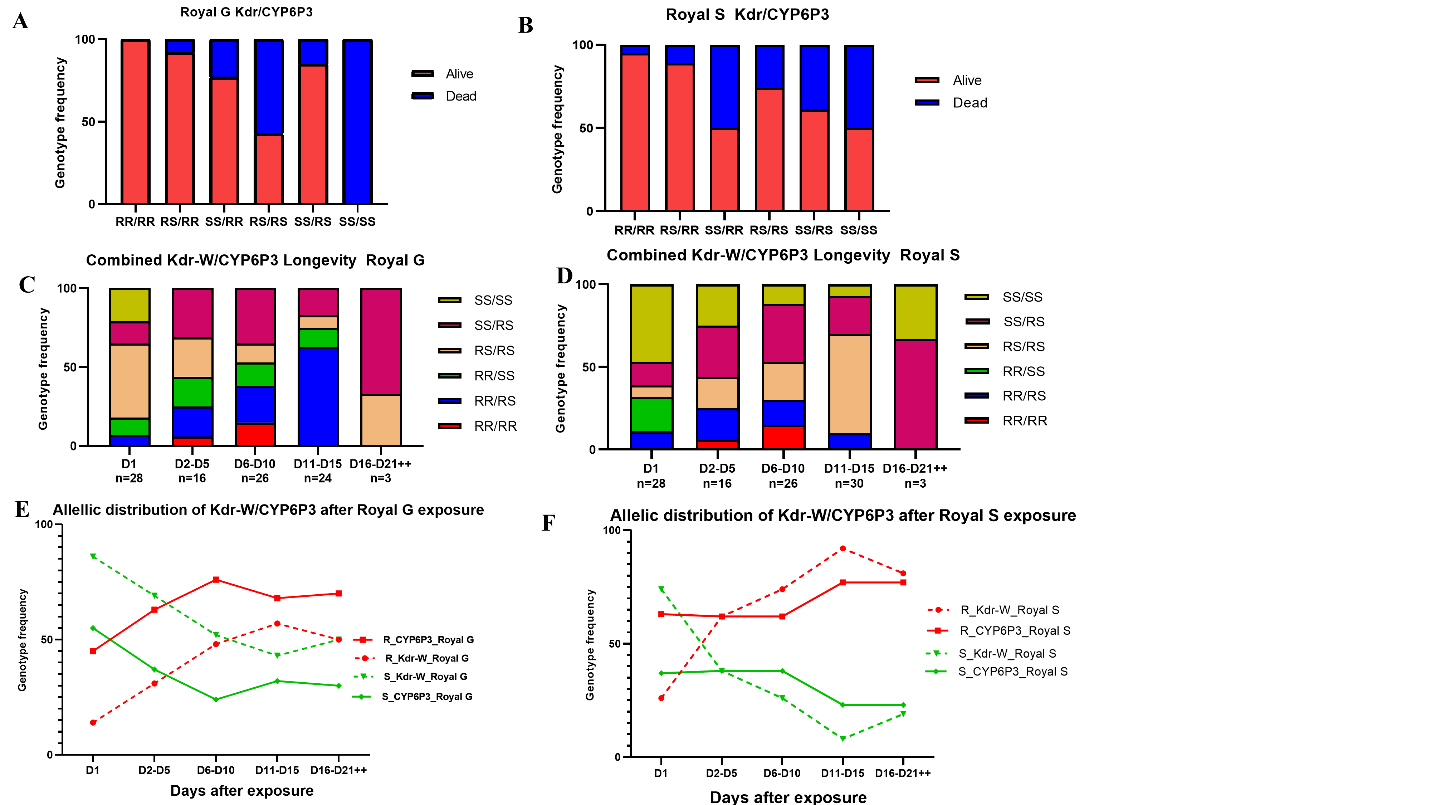


**Figure S3**: Correlation between Kdr-W, CYP6P3, CYP6P9a, CYP6P9b and 6.5Kb-SV resistant marker and the blood feeding ability RG and RS exposure. Figures (A, B) represent the combined effect of the correlation between the L1014F_Kdr-W/G615C_CYP6P3 genotypes and ability to survive to Royal G and (C,E) represent the cumulative effect of on longevity for Royal G and (D,F) to Royal S.


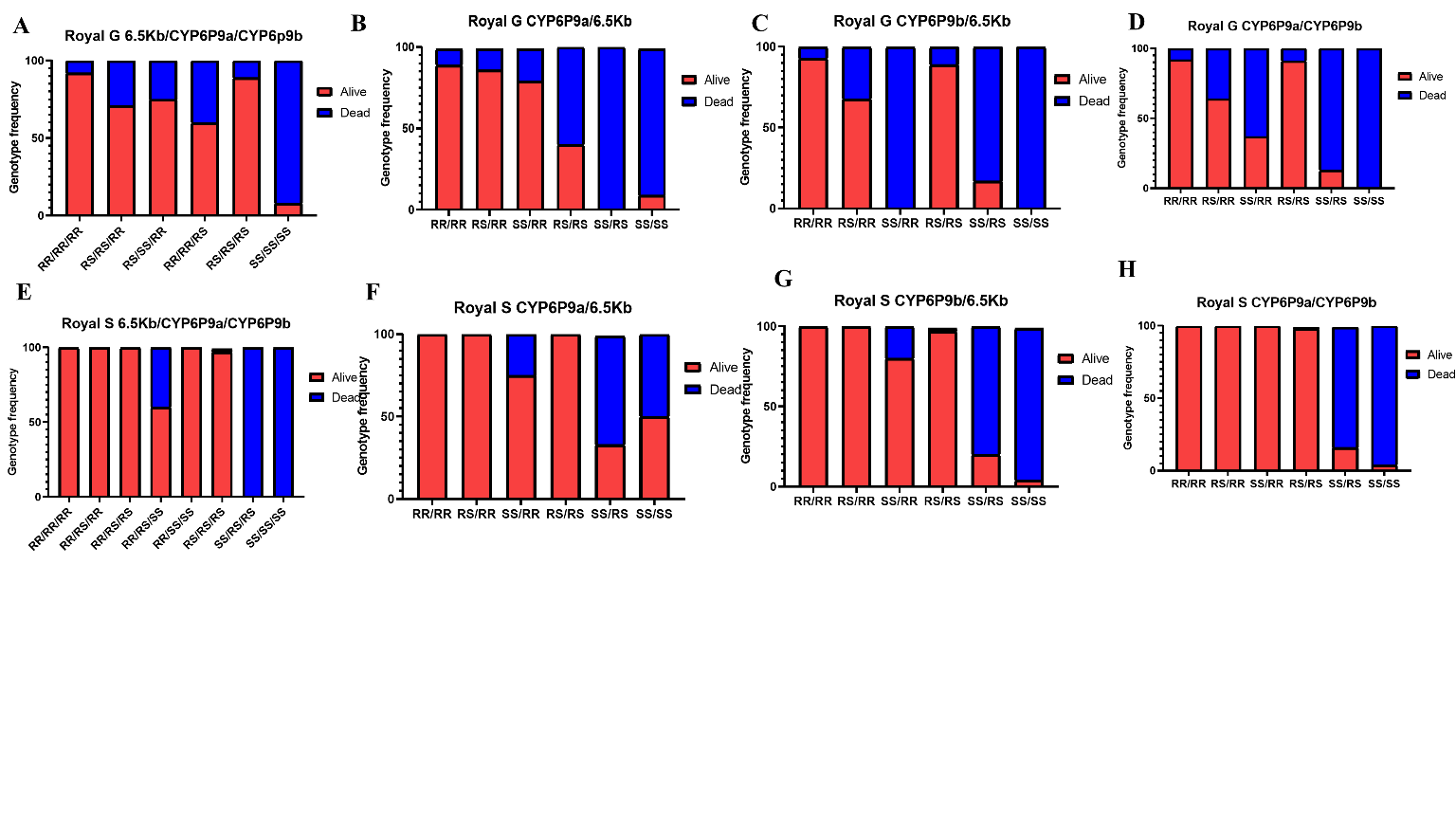


**Figure S4:** Cumulative effect of L1014F_Kdr-W/E205D_CYP6P3 on life traits parameter after exposure to bed nets. Figures (A) represent the combined effect of the three mechanisms CYP6P9a/CYP6P9b/6,5Kb-SV for Royal G, (E) for Royal S; (B, F) the combined effect of two mechanisms CYP6P9a/6.5Kb-SV for Royal G and Royal S; (C,G) the combined effect of two mechanisms CYP6P9b/6.5Kb-SV for Royal G and Royal S; (D,H) the combined effect of two mechanisms CYP6P9a/CYP6P9b for Royal G and Royal S.


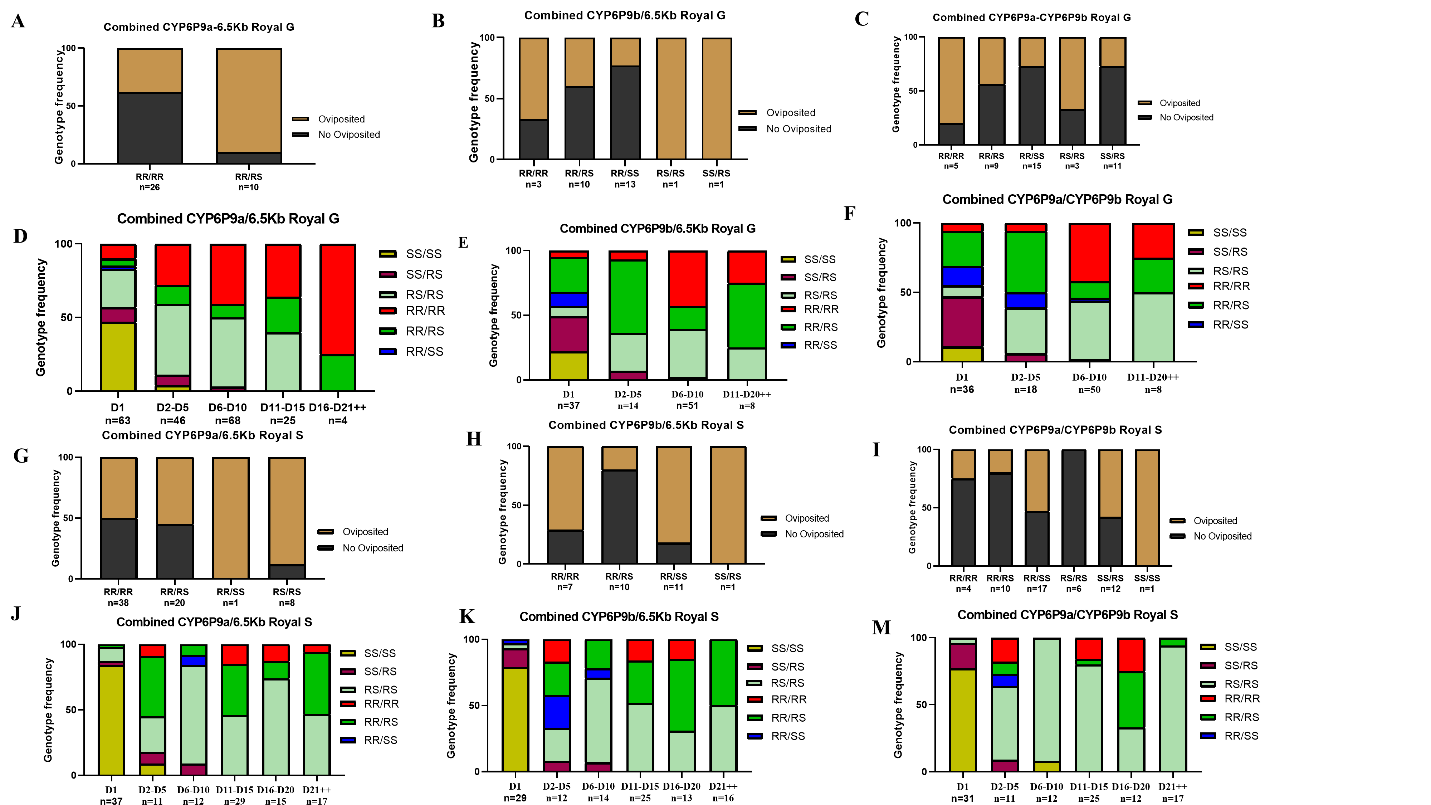


**Figure S5:** Cumulative effect of CYP6P9a, CYP6P9b, and 6,5kb-SV on life traits parameter after exposure to bed nets. Figures (A, D) represent the combined effect of two mechanisms CYP6P9a/6.5Kb-SV for Royal G, (G, J) for Royal Sentry on fecundity and longevity; (B, E) the combined effect of two mechanisms CYP6P9b/6.5Kb-SV for Royal G, (H, K) for Royal Sentry on fecundity and longevity; (C, F) the combined effect of two mechanisms CYP6P9a/CYP6P9b for Royal G, (I, M) for Royal Senty on fecundity and longevity.


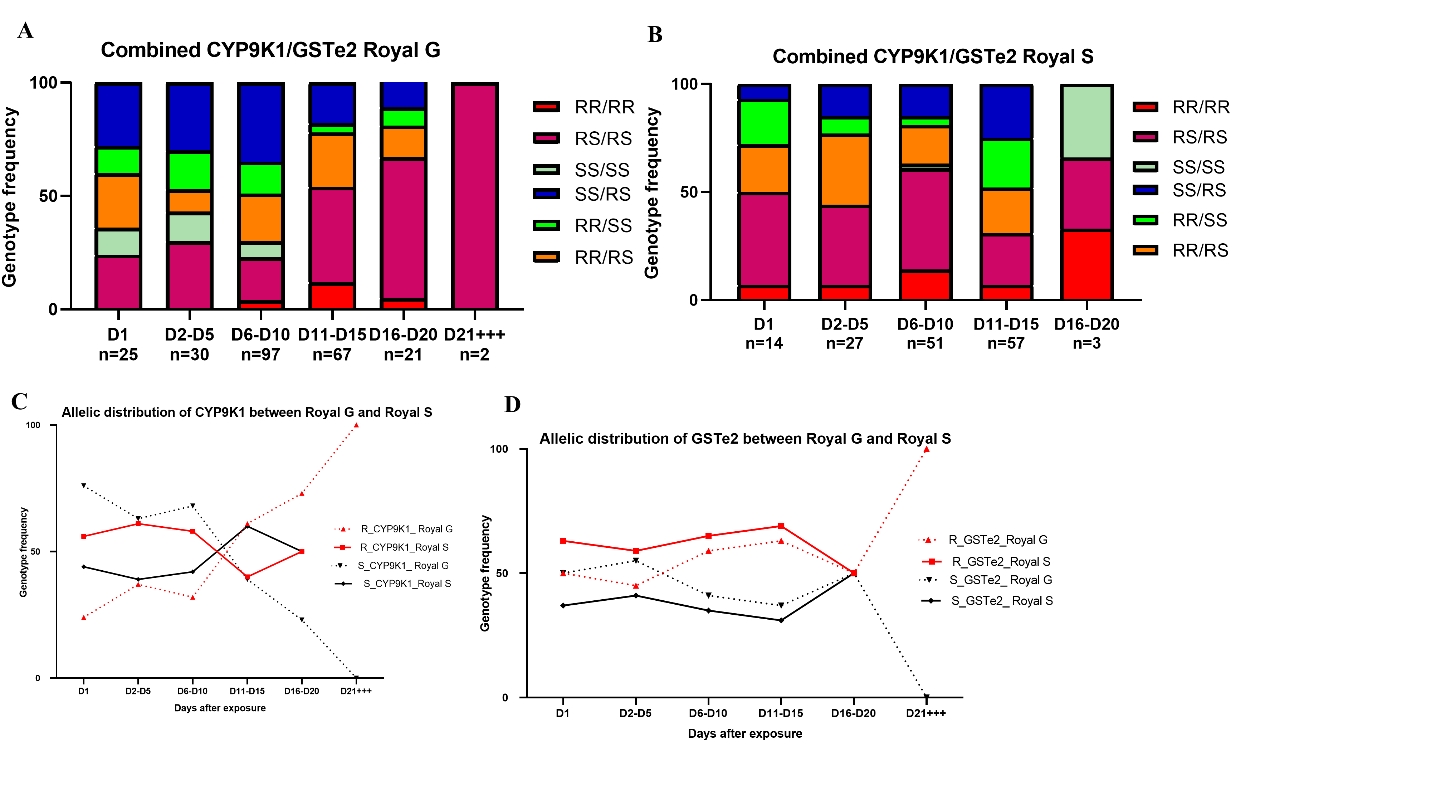


**Figure S6:** Cumulative effect of L119F_GSTe2/G454A_CYP9K1 on life traits parameter after exposure to bed nets. Figures (A, B) represent the combined effect of the correlation between the L119F_GSTe2/G454A_CYP9K1 genotypes on longevity after Royal G and Royal S exposure at the genotypic level; (C, D) at the allelic level.
