# Supplementary material for: Long‐term impact of exposure to Royal Guard, a pyriproxyfen‐based bed net, on pyrethroid‐resistant malaria vectors from Cameroon using DNA‐based metabolic resistance markers
Source: Pest Manag Sci. 2025 Jan 23;81(4):2165–80. doi: 10.1002/ps.8615 (PMC11906912; doi:10.1002/ps.8615)
Supplement: Supplementary file 2 — Table S1. List of primers used in quantitative real‐time PCR. Table S2. List of primers and probes used for the detection of known resistance markers. Table S3. Evaluation of the association between different genotypes of the Kdr‐W, CYP6P3, CYP6P9a, CYP6P9b and 6.5Kb‐SV mutations and female longevity in exposed mosquitoes. Table S4. Evaluation of the association between different genotypes of GSTe2 and CYP9K1 mutation and female longevity in exposed mosquitoes. Table S5. Evaluation of the association between combined genotypes of GSTe2/CYP9K1 mutation and female longevity in exposed mosquitoes. Table S6. Evaluation of the association between combined genotypes of Kdr/CYP6P3 and CYP6P9a/CYP6P9b mutation and female longevity in exposed mosquitoes. Table S7. Correlation between Kdr‐W, CYP6P3, CYP6P9a, CYP6P9b and 6.5Kb‐SV resistant markers and the blood feeding ability of Royal Guard and Royal Sentry exposure. [file PS-81-2165-s002.docx]

| **Gene name** | **qPCR primers** | **Sequences** | **Length (18-24)** | **Tm (50-65°C)** | **GC % (35-65)** | **Product Size (150-200bp)** |
| --- | --- | --- | --- | --- | --- | --- |
|  |  |  |  |  |  |  |
| **Kr-h1** | qPCRAGA009662F1 | TCCGTCATTCAGTACGCAAA | 20 | 60 | 50% | 197bp |
|  | qPCRAGA009662R1 | gcgtgaggttattggttgct | 20 | 60 | 45% |  |
| **EcRA** | qPCRAGAP029539F1 | ACGGGCGTCAGCAGTAATAG | 20 | 60 | 55 | 200bp |
|  | qPCRAGAP029539R1 | gatgcacgtgactgtcgaac | 20 | 60 | 55 |  |
| **HR3** | qPCRAGAP009002F1 | ATGCACATGGAGGCGTTACA | 20 | 60 | 55 | 186bp |
|  | qPCRAGAP009002R1 | ctgctgatggtggtgatggt | 20 | 60 | 50 |  |
| **Met** | qPCRAGAP006022F1 | TGGCACTGCACGATCAGAAA | 20 | 60 | 50 | 168bp |
|  | qPCRAGAP006022R1 | gtagtcggggtagaggggaa | 20 | 59 | 60 |  |
| **20E** | qPCRAGAP029560F1 | AGCAGCAGCAGCAACAATC | 19 | 60.3 | 52.60% | 151bp |
|  | qPCRAGAP029560R1 | ctcccactgatccctctcct | 20 | 60.6 | 60% |  |

**Table S1:** List of primers used in quantitative real-time PCR

**Table S2:** List of primers and probes used for the detection of Known resistance markers

| ***L1014F-Kdr-W*** | Agd1 | ATAGATTCCCCGACCATG |
| --- | --- | --- |
|  | Agd2 | AGACAAGGATGATGAACC |
|  | Agd3 | AATTTGCATTACTTACGACA |
|  | Agd4 | CTGTAGTGATAGGAAATTTA) |
|  | | |
| ***G615C-CYP6P3*** | CYP6p3_1050bp-F | CGTTTTACGACGGACGTGAT |
|  | CYP6p3_1050bp-R | TTCAGCACCCAACGAACGAA |
|  | Probe_WT-HEX | ACTTG+CG+GA+A+C+TC |
|  | Probe_MUT-Fam | AC+TTGCG+GA+A+G+TC |
|  | 9K1OF | 5’-ACTGGACCGATGATGATTTGAC |
| ***G454A-CYP9K1*** | 9K1OR | 5’-ATCCAGAAGCCCTTCTCTGC |
|  | 9K1IF | 5’-GGATCGTTTCTGGCCGGAAGGTTGG**C** |
|  | 9K1IR | 5’-TATCGATCGGT GTCGGGCTGTCCG CT**C** |
| ***L119F-Gste2*** | L119F-Sus | CATTTCTTATTCTCATTTACAGGAGCGTAaTC |
|  | L119F-Res | CGGGAATGTCCGATTTTCCGTAGAAtAA |
|  | Ndel_GSTe2 F | GGAATTCCATATGACCAAGCTAGTTCTGTACACGCT |
|  | Xdal _GSTe2 R | TCTACATCAAGCTTTAGCATTTTCCTCCTT |
|  | | |
| ***CYP6P9a*** | 6P9a1_F | TCCCGAAATACAGCCTTTCAG |
|  | RFLP_6P9a_R | ATTGGTGCCATCGCTAGAAG |
| ***CYP6P9b*** | 6P9bdpL_F | CCCCCACAGGTGGTAACTATCTGAA |
|  | 6P9b_ RFLP_0.5_R | TTATCCGTAACTCAATAGCGATG |
| **6.5 kb** | FG_5_F | CTTCACGTCAAAGTCCGTAT |
|  | FG_3_R | TTTCGGAAAACATCCTCAA |
|  | FZ_INS5_R | ATATGCCACGAAGGAAGCAG |

*F= Forward, R= Reverse, Rsp= Ribosomal protein

| **Genotypes** | **(D1) x ( D2-D5)** | | | **(D1) x ( D6-D10)** | | | **(D1) x ( D11-D15)** | | | **(D1) x ( D16-D21++)** | | |
| --- | --- | --- | --- | --- | --- | --- | --- | --- | --- | --- | --- | --- |
|  | **OR** | **CI** | **P** | **OR** | **CI** | **P** | **OR** | **CI** | **P** | **OR** | **CI** | **P** |
| **Kdr_W** | | | | | | | | | | | | |
| **RR vs SS** | 6.25 | 2.1-17.5 | **0.0005*** | 16.5 | 5.8-44.9 | **<.0001*** | 32.5 | 9.9-90.6 | **<.0001*** | 18.75 | 6.3-50.33 | **<.0001*** |
| **RR vs RS** | 4.1 | 1.3-12.4 | **0.03*** | 10.5 | 3.12-30.5 | **<.0001*** | 3.6 | 1.1-10.35 | **0.03*** | 262.5 | 28.9-2733 | **<0.0001*** |
| **RS vs SS** | 1.5 | 0.77-2.96 | **0.29** | 1.5 | 0.7-3.13 | **0.26** | 9.02 | 4.2-18.6 | **<.0001*** | 14 | 2.33-148 | **0.001*** |
| **R vs S** | 2.76 | 1.33-5.71 | **0.006*** | 5.67 | 2.8-11.4 | **<.0001*** | 8.14 | 4.14-16.4 | **<.0001*** | 6.14 | 3.13-12.3 | **<.0001*** |
| **CYP6P3** | | | | | | | | | | | | |
| **RR vs SS** | 4.6 | 2.01-10.96 | **0.0004*** | 22.6 | 7.1-64.28 | **<.0001*** | 5.8 | 2.4-12.96 | **<.0001*** | 4.9 | 2.2-10.63 | **<.0001*** |
| **RR vs RS** | 2.3 | 1.1-4.6 | **0.01*** | 4.3 | 2.2-8.2 | **<.0001*** | 4.4 | 2.2-8.7 | **<.0001*** | 9.7 | 4.6-19.36 | **<0.0001*** |
| **RS vs SS** | 1.9 | 0.8-4.1 | **0.09** | 5.2 | 1.7-14.5 | **0.002*** | 1.3 | 0.62-2.73 | **0.56** | 1.96 | 0.90-4.31 | **0.11** |
| **R vs S** | 2.08 | 1.17-3.62 | **0.01*** | 3.8 | 2.14-7.07 | **<.0001*** | 2.59 | 1.4-4.6 | **0.0016*** | 2.85 | 1.5-5.17 | **0.0006*** |
| **CYP6P9a** | | | | | | | | | | | | |
| **RR vs SS** | 22.6 | 7.4-59.1 | **<.0001*** | 81.2 | 19.7-270.9 | **<.0001*** | 226.7 | 30.3-2334 | **<.0001*** | 566.7 | 79.6-5756 | **<.0001*** |
| **RR vs RS** | 2.3 | 1.04-5.7 | **0.05** | 3.5 | 1.6-8.4 | **0.001*** | 2.9 | 1.34-7.06 | **0.009*** | 444.4 | 61.3-4537 | **<0.0001*** |
| **RS vs SS** | 9.5 | 4.02-20.9 | **<.0001*** | 22.9 | 6.9-73.01 | **<.0001*** | 76.5 | 13.6-787.3 | **<.0001*** | 1.27 | 0.06-24.6 | **>0.99** |
| **R vs S** | 4.3 | 2.3-7.6 | **<.0001*** | 6.5 | 3.5-12.5 | **<.0001*** | 8.2 | 4.3-15.39 | **<.0001*** | 26.83 | 11.5-57.9 | **<.0001*** |
| **CYP6P9b** | | | | | | | | | | | | |
| **RR vs SS** | 38.9 | 6.3-409.4 | **<.0001*** | 35.1 | 5.6-369.7 | **<.0001*** | 31.9 | 5.1-336.7 | **<.0001*** | - | - | **-** |
| **RR vs RS** | 1.7 | 0.97-3.17 | **0.06** | 1.3 | 0.7-2.5 | **0.29** | 1.13 | 0.62-2.08 | **0.76** | - | - | **-** |
| **RS vs SS** | 21.8 | 3.4-231.8 | **<.0001*** | 25.2 | 4.08-266 | **<.0001*** | 28.05 | 4.5-295.7 | **<.0001*** | - | - | **-** |
| **R vs S** | 3.1 | 1.6-5.7 | **0.0004*** | 108.2 | 32.2-340.3 | **<.0001*** | 097 | 29.2-304.6 | **<.0001*** | - | - | **-** |
| **6.5Kb_SV** | | | | | | | | | | | | |
| **RR vs SS** | 24.5 | 7.9-62.8 | **<.0001*** | 171.5 | 26.8-1762 | **<.0001*** | 196 | 30.9-2010 | **<.0001*** | 290.5 | 46.7-2963 | **<.0001*** |
| **RR vs RS** | 1.02 | 0.47-2.18 | **>0.9** | 1.7 | 0.82-3.5 | **0.20** | 2.2 | 1.06-4.6 | **0.04*** | 8.5 | 3.84-19.4 | **<.0001*** |
| **RS vs SS** | 24 | 8.4-59.04 | **<.0001*** | 100 | 17.1-1030 | **<.0001*** | 88 | 14.9-908.3 | **<.0001*** | 34 | 5.05-362.2 | **<.0001*** |
| **R vs S** | 4.33 | 2.3-7.6 | **<.0001*** | 6.6 | 3.5-12.54 | **<.0001*** | 8.27 | 4.3-15.3 | **<.0001*** | 26.8 | 11.5-57.9 | **<.0001*** |

**Table S3**: Evaluation of the association between different genotypes of the Kdr-W, CYP6P3, CYP6P9a, CYP6P9b and 6.5Kb-SV mutation and female longevity in exposed mosquitoes

CI Confidence interval, D day (post‑exposure), RR homozygous resistant, RS heterozygous resistant; SS homozygous susceptible, OR odds ratio *Indicates significant difference between genotypes for the OR

**Table S4**: Evaluation of the association between different genotypes of GSTe2 and CYP9K1 mutation and female longevity in exposed mosquitoes

| **Genotypes** | **(D1) x ( D2-D5)** | | | **(D1) x ( D6-D10)** | | | **(D1) x ( D11-D15)** | | | **(D1) x ( D16-D20)** | | | **(D1) x ( D21++)** | | |
| --- | --- | --- | --- | --- | --- | --- | --- | --- | --- | --- | --- | --- | --- | --- | --- |
|  | **OR** | **CI** | **P** | **OR** | **CI** | **P** | **OR** | **CI** | **P** | **OR** | **CI** | **P** | **OR** | **CI** | **P** |
| **GSTe2-L119F** | | | | | | | | | | | | | | | |
| **RR vs SS** | 1.6 | 0.7-3.7 | **0.29** | 2.2 | 0.9-5.1 | **0.09** | 2.9 | 1.2-6.4 | **0.01*** | 1 | 0.4-2.3 | **>0.99** | 1 | 0.05-19.7 | **>0.99** |
| **RR vs RS** | 1.4 | 0.7-3.07 | **0.3** | 1.3 | 0.7-2.5 | **0.4** | 1.6 | 0.8-3.1 | **0.14** | 1.5 | 0.7-2.9 | **0.28** | 46.1 | 7.9-481.7 | **<.0001*** |
| **RS vs SS** | 1.1 | 0.5-2.09 | **0.8** | 1.6 | 0.7-3.3 | **0.26** | 1.7 | 0.8-3.7 | **0.18** | 1.5 | 0.7-2.9 | **0.28** | 46.1 | 7.9-481.7 | **<.0001*** |
| **R vs S** | 1.2 | 0.69-2.15 | **0.57** | 1.43 | 0.81-2.4 | **0.25** | 1.7 | 0.96-2.94 | **0.08** | 1 | 0.57-1.75 | **>0.99** | 100 | 16.6-1024 | **<.0001*** |
| **CYP9K1** | | | | | | | | | | | | | | | |
| **RR vs SS** | 1.73 | 0.7-3.9 | **0.26** | 1.3 | 0.57-3.07 | **0.6** | 17.07 | 6.7-41.5 | **<.0001*** | 240 | 35.4-2461 | **<.0001*** | 533.3 | 81.8-5410 | **<.0001*** |
| **RR vs RS** | 1.8 | 0.7-4.4 | **0.23** | 1.6 | 0.64-4.3 | **0.3** | 1.1 | 0.48-2.6 | **0.8** | 1.6 | 0.7-3.7 | **0.24** | 200 | 28.0-2076 | **<0.0001*** |
| **RS vs SS** | 3.1 | 1.6-5.8 | **0.0004*** | 2.2 | 1.5-4.2 | **0.01*** | 15.4 | 6.6-34.6 | **<.0001*** | 146.7 | 24.9-1504 | **<.0.001*** | 2.6 | 0.1-5132 | **0.4** |
| **R vs S** | 1.86 | 1-3.42 | **0.06** | 1.49 | 0.81-2.7 | **0.27** | 4.95 | 2.7-9.1 | **<.0001*** | 10.05 | 5.1-19.49 | **<.0001*** | 316.7 | 55.7-3216 | **<.0001*** |

CI Confidence interval, D day (post‑exposure), RR homozygous resistant, RS heterozygous resistant; SS homozygous susceptible, OR odds ratio *Indicates significant difference between genotypes for the OR

**Table S5:** Evaluation of the association between combined genotypes of GSTe2/CYP9K1 mutation and female longevity in exposed mosquitoes

| **Genotypes** | **(D1) x ( D2-D5)** | | | **(D1) x ( D6-D10)** | | | **(D1) x ( D11-D15)** | | | **(D1) x ( D16-D20)** | | | **(D1) x ( D21++)** | | |
| --- | --- | --- | --- | --- | --- | --- | --- | --- | --- | --- | --- | --- | --- | --- | --- |
|  | **OR** | **CI** | **P** | **OR** | **CI** | **P** | **OR** | **CI** | **P** | **OR** | **CI** | **P** | **OR** | **CI** | **P** |
| **GSTe2-L119F/CYP9K1** | | | | | | | | | | | | | | | |
| **RR/RR vs RS/RR** | 2.4 | 0.11-47.2 | **0.52** | 4.51 | 0.6-57.91 | **0.34** | 12 | 1.7-133 | **0.0091*** | 8.57 | 0.9-104.4 | **0.07** | 24 | 0.64-462.3 | **0.14** |
| **RR/RR vs SS/RR** | 1.41 | 0.06-28.32 | **>0.99** | 3.4 | 0.42-44.97 | **0.36** | 36 | 3.85-409 | **0.0005*** | 7.5 | 0.9-94.4 | **0.16** | 12 | 0.3-240 | **0.25** |
| **RR/RR vs RS/RS** | 1.25 | 0.06-24.45 | **>0.99** | 5.05 | 0.6-63.98 | **0.18** | 6.8 | 1.06-76.26 | **0.05*** | 1.93 | 0.23-23.66 | **>0.99** | 4.16 | 0.21-79.76 | **0.35** |
| **RR/RR vs SS/RS** | 1.07 | 0.05-20.97 | **>0.99** | 3.2 | 0.47-40.3 | **0.38** | 18.67 | 2.67-206 | **0.001*** | 10 | 1.07-121.1 | **0.0298** | 28 | 0.7-536.4 | **0.12** |
| **RR/RR vs SS/SS** | 1.08 | 0.05-22.01 | **>0.99** | 6.85 | 0.7-89.56 | **0.14** | 144 | 7.05-1594 | **<.0001*** | 60 | 3.1-732.5 | **0.002*** | 12 | 0.32-240 | **0.25** |
| **RS/RS vs SS/SS** | 1.15 | 0.43-2.99 | **0.81** | 1.35 | 0.46-3.89 | **0.78** | 21 | 3.2-229.6 | **0.0004*** | 31 | 4.8-336.5 | **<.0001*** | 50 | 8.01-539.8 | **<.0001*** |

CI Confidence interval, D day (post‑exposure), RR homozygous resistant, RS heterozygous resistant; SS homozygous susceptible, OR odds ratio *Indicates significant difference between genotypes for the OR

**Table S6**: Evaluation of the association between combined genotypes of Kdr/CYP6P3 and CYP6P9a/CYP6P9b mutation and female longevity in exposed mosquitoes

| **Genotypes** | **(D1) x ( D2-D5)** | | | **(D1) x ( D6-D10)** | | | **(D1) x ( D11-D15)** | | | **(D1) x ( D16-D21++)** | | |
| --- | --- | --- | --- | --- | --- | --- | --- | --- | --- | --- | --- | --- |
|  | **OR** | **CI** | **P** | **OR** | **CI** | **P** | **OR** | **CI** | **P** | **OR** | **CI** | **P** |
| **Kdr-W/CYP6P3** | | | | | | | | | | | | |
| **RR/RR vs RS/RR** | 2.2 | 0.2-28 | **0.65** | 4.5 | 0.6-54.4 | **0.22** | 9 | 0.41-172 | **0.21** | 7 | 0.1-147.6 | **0.37** |
| **RR/RR vs SS/RR** | 3.47 | 0.40-43.07 | **0.38** | 11 | 1.35-125.4 | **0.01*** | 1.09 | 0.05-22.28 | **>0.99** | 11 | 0.29-221 | **0.27** |
| **RR/RR vs RS/RS** | 11.2 | 1.6-131.7 | **0.01*** | 58.75 | 8.6-629 | **<.0001*** | 0.17 | 0.008-3.61 | **0.29** | 1.42 | 0.07-27.5 | **>0.99** |
| **RR/RR vs SS/RS** | 2.7 | 0.3-33.09 | **0.65** | 6 | 0.8-67.4 | **0.09*** | 1.21 | 0.06-24.28 | **>0.99** | 4.78 | 0.23-91.91 | **0.33** |
| **RR/RR vs SS/SS** | 126 | 7.7-1450 | **<.0001*** | 315 | 17.6-3328 | **<.0001*** | 21 | 0.56-406.8 | **0.16** | 21 | 0.56-406.8 | **0.16** |
| **RS/RS vs SS/SS** | 11.17 | 1.6-120 | **0.005*** | 5.3 | 0.8-59.9 | **0.10** | 3.5 | 0.5-41.3 | **0.43** | 14.7 | 2.3-157.5 | **0.0007*** |
| **CYP6P9a/ CYP6P9b** | | | | | | | | | | | | |
| **RR/RR vs RS/RR** | 1.76 | 0.46-6.26 | **0.52** | 14.5 | 4.9-46.24 | **<.0001*** | 4.16 | 1.5-12.5 | **0.009*** | . | . | **.** |
| **RR/RR vs SS/RR** | 1.27 | 0.35-4.54 | **>0.99** | 49 | 9.2-232.4 | **<.0001*** | 58.3 | 6.5-634 | **<.0001*** | . | . | **.** |
| **RR/RR vs RS/RS** | 4.12 | 1.14-14.72 | **0.05*** | 1.33 | 0.44-4.37 | **0.77** | 1.5 | 0.4-4.38 | **0.54** | . | . | **.** |
| **RR/RR vs SS/RS** | 6 | 1.603-24.92 | **0.01*** | 126 | 23.2-569 | **<.0001*** | 150 | 19.4-1571 | **<.0001*** | . | . | **.** |
| **RR/RR vs SS/SS** | 11 | 1-135 | **0.06** | 77 | 8.4-839.5 | **<.0001*** | 42.8 | 6.15-507.8 | **<.0001*** | . | . | **.** |
| **RS/RS vs SS/SS** | 45.3 | 5.38-499 | **<.0001*** | 57.7 | 6.9-631.4 | **<.0001*** | 68.75 | 8.3-749.1 | **<.0001*** | . | . | **.** |

CI Confidence interval, D day (post‑exposure), RR homozygous resistant, RS heterozygous resistant; SS homozygous susceptible, OR odds ratio *Indicates significant difference between genotypes for the OR

**Table S7:** Correlation between Kdr-W, CYP6P3, CYP6P9a, CYP6P9b and 6.5Kb-SV resistant marker and the blood feeding ability Royal G and Royal S exposure.

| **Kdr-W** | | | | | | |
| --- | --- | --- | --- | --- | --- | --- |
|  | **Royal G** | | | **Royal S** | | |
|  | **OR** | **CI** | **P** | **OR** | **CI** | **P** |
| **RR vs SS** | 1.37 | 0.47-4.2 | **0.56** | 3.22 | 0.85-10.04 | **0.07** |
| **RR vs RS** | 1.01 | 0.37-2.87 | **>0.99** | 1.95 | 0.56-5.98 | **0.38** |
| **RS vs SS** | 1.36 | 0.74-2.5 | **0.36** | 1.65 | 0.91-3.04 | **0.10** |
| **R vs S** | 1.18 | 0.67-2.08 | **0.66** | 1.55 | 0.87-2.82 | **0.18** |
|  | | | | | | |
| **CYP6P3** | | | | | | |
|  | **Royal G** | | | **Royal S** | | |
|  | **OR** | **CI** | **P** | **OR** | **CI** | **P** |
| **RR vs SS** | 2.01 | 0.74-5.57 | **0.21** | 0.63 | 0.22-1.89 | **0.57** |
| **RR vs RS** | 1.31 | 0.72-2.43 | **0.44** | 1.06 | 0.55-2.02 | **0.86** |
| **RS vs SS** | 1.53 | 0.60-4 | **0.47** | 0.60 | 0.23-1.67 | **0.45** |
| **R vs S** | 1.35 | 0.77-2.40 | **0.37** | 0.92 | 0.51-1.63 | **0.88** |
|  | | | | | | |
| **CYP6P9a** | | | | | | |
|  | **Royal G** | | | **Royal S** | | |
|  | **OR** | **CI** | **P** | **OR** | **CI** | **P** |
| **RR vs SS** | 2.2 | 0.97-5.20 | **0.06** | 3.8 | 1.22-10.42 | **0.01*** |
| **RR vs RS** | 1.01 | 0.49-2.16 | **>0.99** | 1.87 | 0.79-4.26 | **0.15** |
| **RS vs SS** | 2.25 | 1.17-4.28 | **0.01*** | 2.02 | 0.88-4.57 | **0.10** |
| **R vs S** | 0.63 | 0.37-1.12 | **0.15** | 1.49 | 0.85-2.65 | **0.20** |
|  | | | | | | |
| **CYP6P9b** | | | | | | |
|  | **Royal G** | | | **Royal S** | | |
|  | **OR** | **CI** | **P** | **OR** | **CI** | **P** |
| **RR vs SS** | 1.14 | 0.05-22.19 | **>0.99** | 2.42 | 0.75-7.27 | **0.16** |
| **RR vs RS** | 1.27 | 0.72-2.27 | **0.47** | 1.38 | 0.74-2.69 | **0.33** |
| **RS vs SS** | 1.11 | 0.05-21.47 | **>0.99** | 3.37 | 0.89-10.55 | **0.07** |
| **R vs S** | 1.22 | 0.67-2.24 | **0.63** | 1.28 | 0.71-2.22 | **0.47** |
|  | | | | | | |
| **6.5Kb-SV** | | | | | | |
|  | **Royal G** | | | **Royal S** | | |
|  | **OR** | **CI** | **P** | **OR** | **CI** | **P** |
| **RR vs SS** | 25.34 | 4.28-272.5 | **<0.0001** | 3.35 | 1.28-8.47 | **0.01** |
| **RR vs RS** | 1.82 | 1.01-3.22 | **0.05** | 2.64 | 1.44-4.79 | **0.001** |
| **RS vs SS** | 13.93 | 2.12-149.8 | **0.002** | 1.26 | 0.46-3.25 | **0.80** |
| **R vs S** | 2.14 | 1.93-3.85 | **0.01** | 2.26 | 1.20-4.28 | **0.01** |
